# Triptycene‐Based Organic Nanosheet Assemblies via Reversible Boronic Ester Formation: Alkyl Chain Length‐Dependent Formation and Aggregation

**DOI:** 10.1002/cplu.70276

**Published:** 2026-10-27

**Authors:** Hironobu Hayashi, Kizuku Yamamoto, Madhuparna Chakraborty, Junko Aimi, Nobuyuki Ishida, Naoki Aratani, Hiroko Yamada

**Affiliations:** ^1^ Center for Basic Research on Materials National Institute for Materials Science (NIMS) Tsukuba Ibaraki Japan; ^2^ Division of Materials Science Nara Institute of Science and Technology (NAIST) Ikoma Nara Japan; ^3^ Research Center for Macromolecules and Biomaterials National Institute for Materials Science (NIMS) Tsukuba Ibaraki Japan; ^4^ Institute for Chemical Research Kyoto University Uji Kyoto Japan

**Keywords:** atomic force microscopy, boronic ester, nanosheet, reversible bond formation, triptycene

## Abstract

A bottom‐up strategy for the preparation of triptycene‐based organic nanosheet assemblies through reversible boronic ester formation is presented. The triptycene scaffold enables the orthogonal placement of alkyl substituents and boronic ester‐forming sites, providing a molecular platform to examine how peripheral alkyl chains influence nanosheet formation and aggregation. Time‐dependent microscopy observations revealed alkyl‐chain‐length‐dependent assembly behavior. Hexyl‐substituted precursors afforded sheet‐like domains at an earlier stage, whereas methyl‐ and ethyl‐substituted analogs showed slower formation of less‐defined nanosheet‐like morphologies. These results indicate that longer alkyl chains are associated with earlier formation of sheet‐like domains and stronger aggregation; however, this alkyl‐chain effect is described as an empirical trend rather than a definitively established mechanism. Infrared spectroscopy supported boronic ester formation, and single‐crystal structures of partial molecular analogs confirmed the orthogonal arrangement of the alkyl substituents relative to the framework‐forming units. Absorption measurements showed a slight red‐shift for the nanosheet assemblies relative to the corresponding reference compounds, consistent with modest extension of electronic communication within the boronic ester‐linked framework. This study provides a case study showing that peripheral alkyl substituents can influence the formation and aggregation behavior of triptycene‐based organic nanosheet assemblies, offering useful insight into the development of dispersible 2D organic materials.

## Graphical abstract

A triptycene‐based platform for dispersible organic nanosheet assemblies was developed using reversible boronic ester formation. Time‐dependent microscopy revealed that peripheral alkyl chains influence sheet‐like domain formation and aggregation behavior: hexyl‐substituted precursors formed sheet‐like domains earlier, whereas shorter alkyl substituents resulted in slower and more defective morphology development. These findings offer useful insight into substituent effects in triptycene‐based 2D organic assemblies.

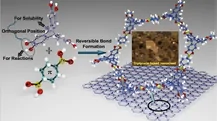

## 1 Introduction

Porous organic materials constructed from discrete molecular building blocks have emerged as highly versatile platforms because their molecular structures, connectivity, and periodicity can be tuned through rational monomer design [1, 2, 3]. In particular, covalent organic frameworks (COFs), which exploit dynamic covalent bonds such as boronic esters and imine linkages, have significantly expanded this field by enabling the construction of robust yet structurally diverse architectures [2, 3, 4, 5, 6, 7, 8]. Among these materials, 2D COFs and related organic frameworks are particularly attractive because their anisotropic structures and large interfacial areas provide opportunities for functional films, membranes, and substrate‐supported materials [2, 3, 4, 5, 6, 7, 8]. However, practical implementation of COFs and related materials is often limited by their generally poor solubility and dispersibility, which directly restricts solution processing and hampers their integration into device‐oriented platforms. Therefore, the development of solution‐processable or dispersible 2D organic materials remains an important challenge [9, 10, 11, 12, 13, 14, 15, 16].

To address these limitations, several strategies have been explored, including mechanical and chemical exfoliation [17], ionic functionalization [11, 18], and the incorporation of bulky substituents [13, 19] to suppress interlayer interactions. For instance, ionic 2D COF nanosheets can remain dispersed in aqueous media through electrostatic repulsion [18], while sterically demanding substituents can facilitate exfoliation or suppress aggregation [19]. Nevertheless, the direct solution‐phase construction of organic nanosheet assemblies with controlled morphology remains insufficiently developed, particularly with respect to how peripheral substituents affect nucleation, aggregation, and sheet‐like assembly during reversible covalent formation.

Previous studies have shown that peripheral substituents and side‐chain interactions can influence the formation, crystallinity, and stacking structures of 2D COFs and related 2D polymers [20, 21, 22, 23]. For example, pendant alkyl or alkoxy chains have been reported to affect stacking geometry, crystallinity, and framework formation in 2D COFs. Related studies have also shown that side‐chain composition can modulate the stacking order and material quality of 2D polymers. These reports indicate that alkyl chains are not merely solubilizing groups but can participate in the organization of layered organic frameworks. Therefore, the effect of alkyl chains on COF and related framework formation has been explored in several systems. However, how peripheral alkyl substituents influence the time‐dependent formation and aggregation of triptycene‐based dispersible nanosheet assemblies remains to be clarified.

Herein, a synthetic approach to triptycene‐based organic nanosheet assemblies formed through reversible boronic ester bonds is presented (Figure 1) [24, 25, 26]. The triptycene scaffold allows the orthogonal introduction of alkyl substituents and network‐forming diol units, providing a molecular platform in which substituent effects can be examined independently from the boronic ester‐forming sites [27]. While previous studies on related triptycene‐based nanosheets mainly employed methyl‐substituted precursors [27], the influence of longer peripheral alkyl chains on the time‐dependent formation and aggregation behavior of such nanosheet assemblies has not been systematically examined. In this study, methyl‐, ethyl‐, and hexyl‐substituted triptycene precursors were compared to examine how alkyl chain length affects the formation, aggregation behavior, and sheet‐like morphology of triptycene‐based nanosheet assemblies. By combining time‐resolved electron microscopy, atomic force microscopy (AFM), infrared (IR) spectroscopy, and crystallographic analysis of partial molecular analogs, this work provides a case study showing that peripheral alkyl substituents can influence the assembly behavior of triptycene‐based dispersible organic nanosheets.

**FIGURE 1:**
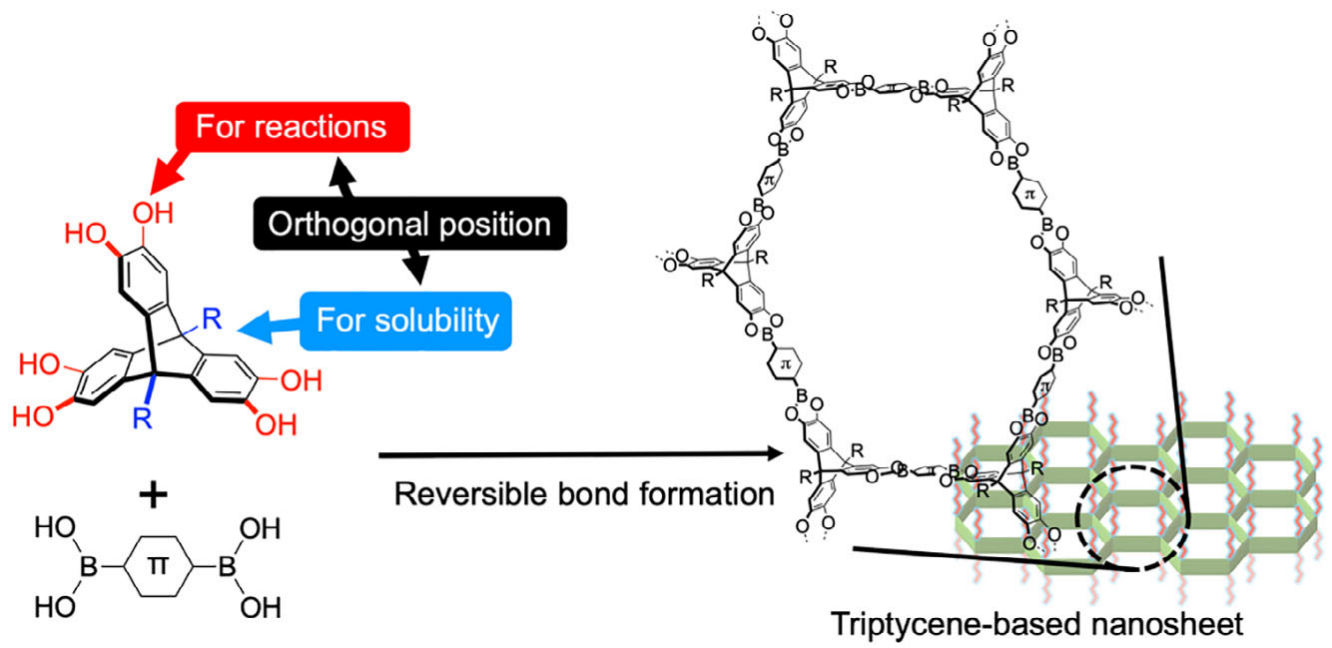
Synthetic strategy for triptycene‐based nanosheet formation.

## 2 Results and Discussion

Hexahydroxylated triptycene derivatives bearing different alkyl substituents as solubilizing groups (**1a**–**c**) were prepared according to established procedures (Scheme 1) [28, 29]. Briefly, demethylation of the corresponding hexamethoxy‐triptycene derivatives using BBr_3_ afforded the triptycene‐hexaol derivatives, which were used as key building blocks for nanosheet assembly. To gain structural insight into the local boronic ester motifs, partial molecular analogs were synthesized by condensing **1a** and **1c** with phenylboronic acid in methanol, followed by solvent removal and thermal annealing at 90 °C under vacuum, affording **TripPh‐Me** and **TripPh‐Hx**, respectively. In addition, **Ref‐L** [30] was used as a linker analog to evaluate the structural characteristics of the phenylene boronic ester unit.

**SCHEME 1:**
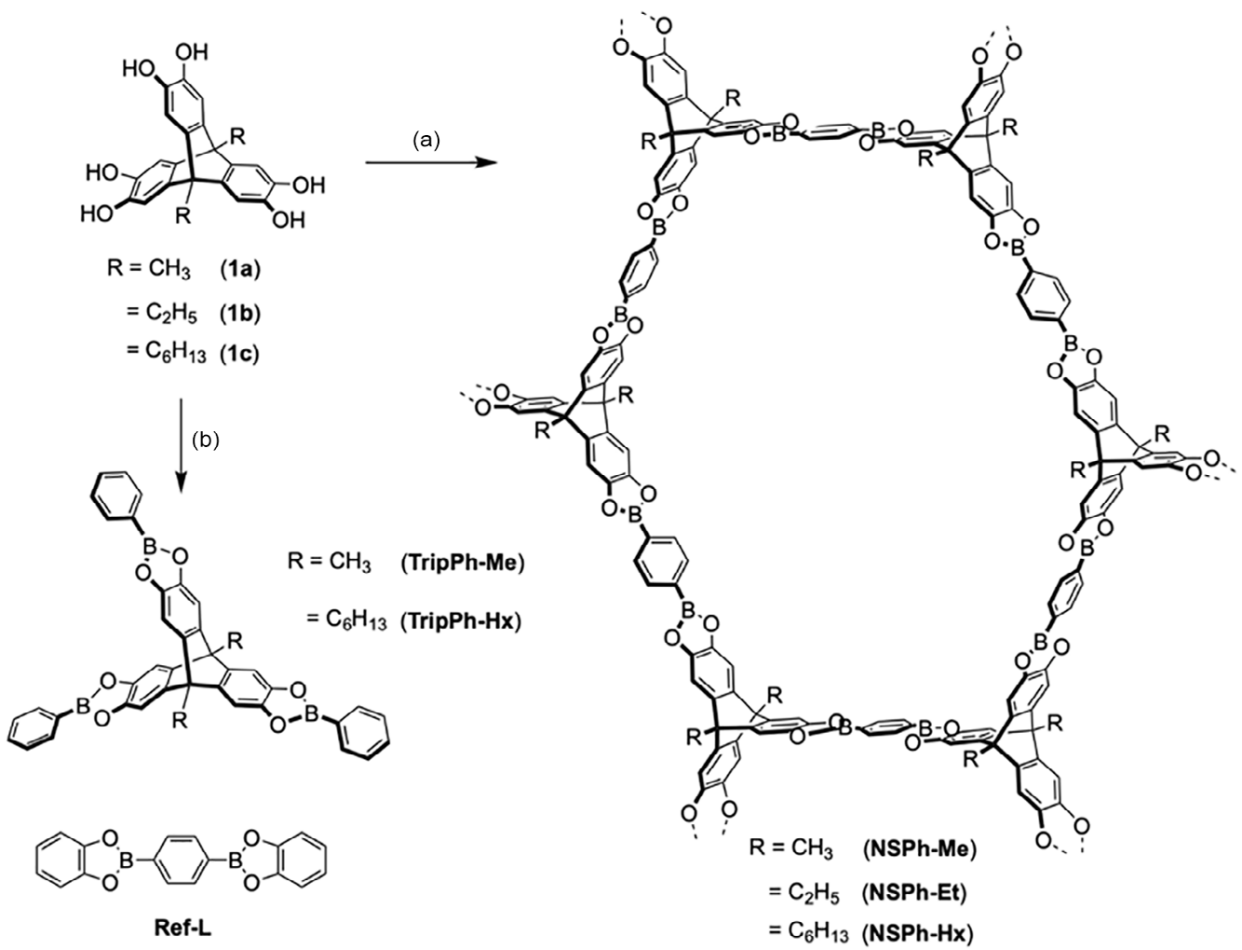
Synthetic scheme for triptycene‐based nanosheets with phenylene linkers and their reference compounds. Conditions: (a) phenyl 1,4‐diboronic acid, mesitylene/1,4‐dioxane (1:1, v/v), 85 °C; (b) phenyl boronic acid, 90 °C, 57% for **TripPh‐Me** and 69% for **TripPh‐Hx**.

Single crystals of these reference compounds were obtained by slow diffusion of acetonitrile into dichloromethane solutions (Figures 2 and S1). Single‐crystal X‐ray diffraction analysis revealed that both **TripPh‐Me** and **TripPh‐Hx** adopt highly symmetric structures, with dihedral angles of close to 120° between the triptycene core and the appended phenyl boronic ester units. The phenyl boronic ester groups are oriented nearly parallel to the phenyl rings of the triptycene core, while the alkyl substituents are arranged approximately orthogonal to the boronic ester‐forming units. Importantly, no substantial differences in the local framework geometry were observed between the methyl‐ and hexyl‐substituted analogs (Figures 2 and S1), indicating that variation of the alkyl chain length does not significantly perturb the triptycene‐based boronic ester motif. These structural features support a molecular design in which alkyl substituents are introduced without directly interfering with the network‐forming sites. The planar structure of **Ref‐L** (Figure 2), together with the 3D triptycene core, suggests that the resulting framework contains aromatic pore walls while reducing close face‐to‐face stacking of the nanosheet assemblies.

**FIGURE 2:**
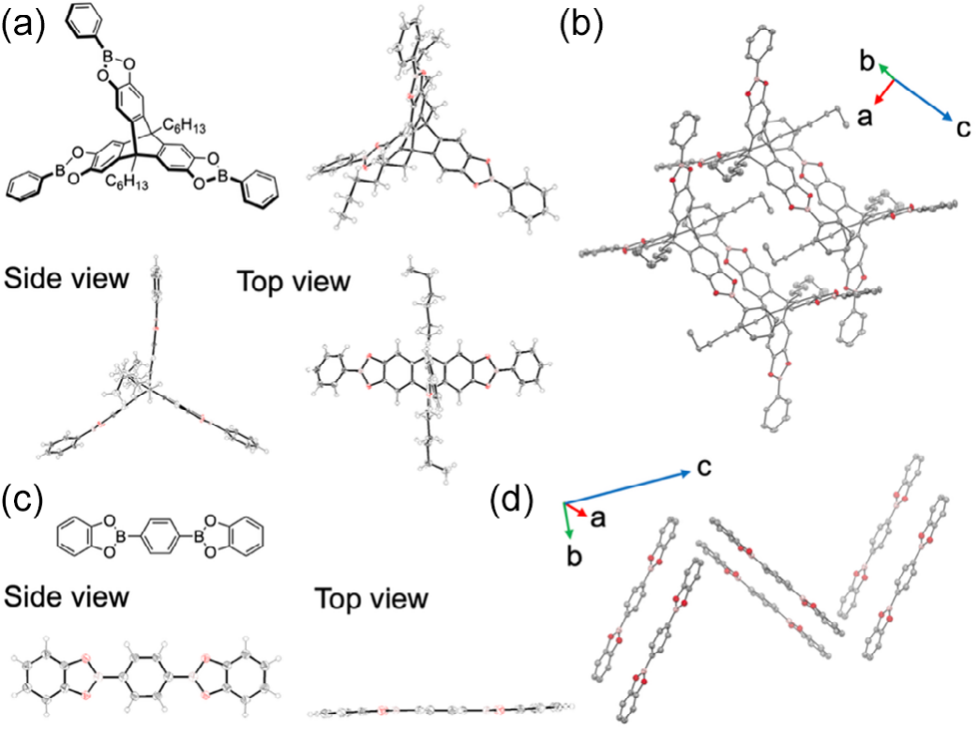
Single‐crystal X‐ray structures of (a) **TripPh‐Hx** and (c) **Ref‐L**. Thermal ellipsoids represent 50% probability. Packing structure of (b) **TripPh‐Hx** and (d) **Ref‐L**. Hydrogen atoms are omitted for clarity.

Triptycene‐based nanosheet assemblies were then prepared through reversible boronic esterification. As a representative example, the formation of **NSPh‐Hx** from hexyl‐substituted triptycene precursor (**1c**) and phenyl 1,4‐diboronic acid was monitored over time. After 12 h, scanning transmission electron microscopy (STEM) already revealed micrometer‐scale sheet‐like features, indicating that substantial growth and assembly had occurred by this time point rather than simple nucleation (Figure 3a). However, the morphology was still developing, as the observed domains were less clearly defined than those obtained after longer reaction times. Extending the reaction to 24 h afforded clearer sheet‐like domains, as further supported by AFM, which revealed multilayered nanosheet assemblies with micrometer‐scale lateral dimensions and thickness of ~4–6 nm in some regions (Figure 3b). This time‐dependent observation suggested that the hexyl‐substituted system undergoes relatively rapid formation of connected sheet‐like domains, followed by further growth and aggregation upon prolonged reaction. After 72 h, scanning electron microscopy (SEM) showed extensive aggregation into densely stacked assemblies. High‐magnification STEM imaging was also performed on relatively thicker regions to examine the structural features more closely (Figure S2). AFM analysis of a toluene‐dispersed sample after drop‐casting confirmed the presence of multilayered nanosheet‐like structures (Figure S3), suggesting that partial redispersion of the aggregated material is possible under appropriate conditions. The theoretical monolayer thickness was estimated from the molecular dimensions obtained from the single‐crystal X‐ray structure of the model compound (Figure 2). When the peripheral hexyl chains are considered, the expected monolayer thickness is ~1.5–2.0 nm. Although the actual conformation of the alkyl chains on mica may differ from the fully extended structure, the experimentally observed height of approximately 2 nm is reasonably consistent with a monolayer or near‐monolayer nanosheet. However, larger domains often exhibited nonuniform heights, suggesting partial stacking, overlap, or aggregation rather than the formation of uniformly stacked, large monolayer nanosheets. This tendency is likely related to the aggregation‐prone nature of the nanosheet assemblies. Therefore, these AFM features are described as stacked or assembled nanosheet‐like domains with micrometer‐scale lateral dimensions in some regions, rather than isolated monolayer nanosheets extending over several micrometers. Control experiments using either **1c** or phenyl 1,4‐diboronic acid alone under identical conditions produced only irregular, nonsheet‐like aggregates (Figure 3c), highlighting the importance of both triptycene‐based precursor design and boronic ester formation for the generation of sheet‐like assemblies. In addition, increasing the precursor concentration led to enhanced random coupling and aggregation, presumably due to increased inter‐nanosheet interactions. This observation emphasizes the importance of carefully controlled assembly conditions.

**FIGURE 3:**
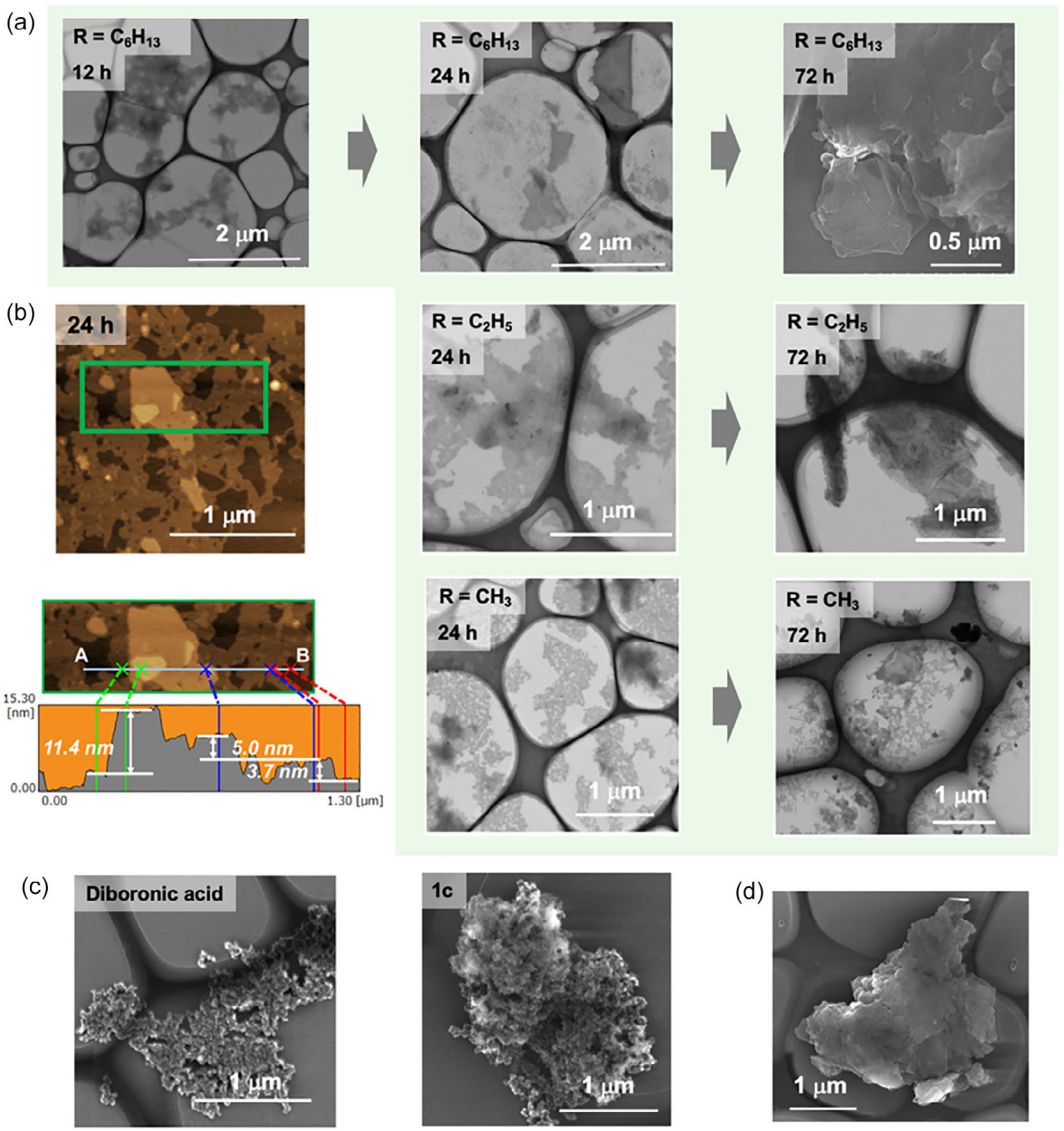
(a) Time‐resolved STEM characterization of **NSPh‐Hx** (top), **NSPh‐Et** (middle), and **NSPh‐Me** (bottom) formation. SEM image is shown for **NSPh‐Hx** after 72‐h reaction. (b) AFM image and height profile of **NSPh‐Hx** after 24‐h reaction. (c) SEM image of aggregations obtained only with phenyl 1,4‐diboronic acid (left) and **1c** (right). (d) SEM image of aggregated **NSPh‐Me** after 72‐h reaction.

The nanosheets were isolated by centrifugation, and were further characterized by IR spectroscopy (Figure 4). For **NSPh‐Hx**, the O─H stretching bands at ~3200–3600 cm^–1^ were markedly reduced relative to the skeletal vibration regions, while new bands appeared around 1270 and 1350 cm^–1^, assignable to C─O and B─O stretching vibrations, respectively [30]. A band in the fingerprint region around ~650 cm^–1^ and the comparable IR features of the reference compound **TripPh‐Hx** also support the formation of boronic ester linkages in **NSPh‐Hx**. The residual O─H signal observed for **NSPh‐Hx** is likely attributable to terminal hydroxy groups at sheet edges or defect sites. Taken together with the microscopy observations (Figure 3), these spectroscopic results support the formation of boronic ester‐linked nanosheet assemblies.

**FIGURE 4:**
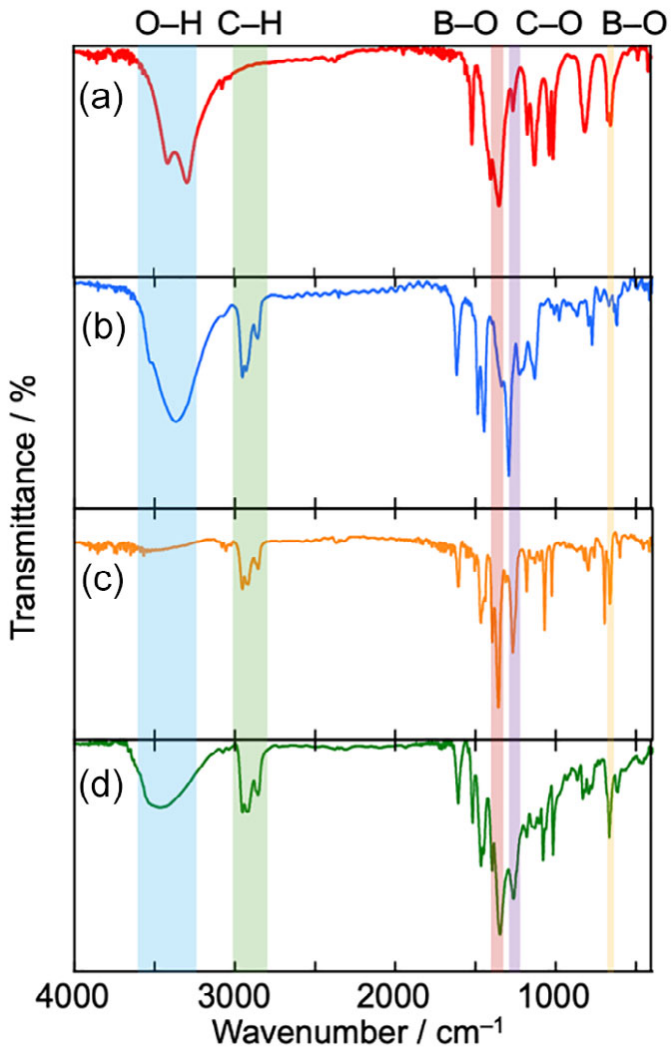
IR spectrum of (a) phenyl 1,4‐diboronic acid, (b) **1c**, (c) **TripPh‐Hx**, and (d) **NSPh‐Hx**.

After confirming boronic ester formation, the influence of alkyl chain length on the nanosheet assembly process was examined, particularly with respect to aggregation behavior relevant to morphology development. This point is important because the present triptycene‐based precursors differ from conventional planar COF building blocks: the 3D triptycene core bears alkyl substituents that can modulate both dispersibility and intermolecular association. To evaluate the effect of alkyl chain length, nanosheet formation was compared using methyl‐ (**1a**), ethyl‐ (**1b**), and hexyl‐substituted (**1c**) triptycene precursors, affording **NSPh‐Me**, **NSPh‐Et**, and **NSPh‐Hx**, respectively. Time‐course microscopy revealed marked differences among the three systems (Figure 3). After 24 h, **NSPh‐Me** and **NSPh‐Et** showed incomplete or ill‐defined nanosheet‐like domains, indicating that the formation of coherent sheet‐like structures was still in progress. In contrast, **NSPh‐Hx** had already formed relatively clear sheet‐like structures. Notably, **NSPh‐Me** exhibited a higher degree of structural defects than **NSPh‐Et**, suggesting that shorter alkyl substituents are associated with slower development of coherent sheet‐like morphology. After 72 h, both **NSPh‐Me** and **NSPh‐Et** eventually afforded nanosheet‐like structures; however, **NSPh‐Me** still contained defective regions, whereas **NSPh‐Et** displayed more developed laminar features. In contrast, **NSPh‐Hx** domains underwent more pronounced aggregation after prolonged reaction, indicating that the longer alkyl chains are associated with stronger aggregation of the nanosheet assemblies. Aggregate structures were rarely observed in the **NSPh‐Me** system, even after extended reaction times (Figure 3d).

In addition to morphological differences, macroscopic observations of the reaction mixtures after 72 h also showed alkyl‐chain‐length‐dependent behavior (Figure S4). The **NSPh‐Me** reaction mixture turned yellow, implying partial oxidation of the triptycene‐hexaol precursor **1a** during the slower assembly process (Figure S5) [31]. In contrast, the **NSPh‐Et** and **NSPh‐Hx** systems retained their original appearance and produced white‐gray precipitates, consistent with more efficient formation of nanosheet assemblies. The recovered mass ratios of the precipitates, calculated from the recovered solid mass relative to the total mass of the starting materials, were 0.61 for **NSPh‐Me**, 0.67 for **NSPh‐Et**, and 0.89 for **NSPh‐Hx**, respectively (see Supporting Information). These values indicate that the amount of aggregated material increases with alkyl chain length. Although these recovered mass ratios should not be interpreted as chemical yields, they provide a useful comparative measure of the extent of aggregated assembly formation under the present conditions.

These observations indicate that alkyl chain length influences the formation and aggregation behavior of triptycene‐based nanosheet assemblies. The earlier appearance of sheet‐like domains in the hexyl‐substituted system can be empirically interpreted as being related to a greater tendency toward intermolecular association during the assembly process [32, 33]. This view is consistent with the crystal packing of **TripPh‐Hx** (Figures 2 and S1), where the hexyl chains are located in close proximity, as well as with the additional AFM observations of **TripPh‐Hx** and **TripPh‐Me** (Figure S6). However, because no direct measurements or molecular‐level simulations of the assembly process were performed, van der Waals interactions or interdigitation cannot be assigned as the definitive origin of the faster nanosheet formation. Thus, the alkyl‐chain effect is best described as an empirical trend: longer alkyl substituents correlate with earlier formation of sheet‐like domains and stronger aggregation, whereas shorter substituents are associated with slower and more defective nanosheet development.

The optical absorption properties of the triptycene‐based nanosheet assemblies were examined and compared with those of the corresponding reference compounds, **TripPh‐Hx** and **Ref‐L** (Figure 5a). Because boronic ester bonds are reversible and sensitive to protic solvents and moisture, freshly prepared samples were used for optical measurements in anhydrous solvents. Protic solvents such as methanol induced rapid decomposition into the corresponding diboronic acid and triptycene precursors (Figure S7), and gradual hydrolysis was also observed in the presence of moisture. In dry 1,4‐dioxane, **TripPh‐Hx** and **Ref‐L** exhibited absorption maxima at 281 nm and 301 nm, respectively. In comparison, **NSPh‐Hx** showed a slightly red‐shifted absorption maximum at 305 nm, consistent with modest extension of electronic communication within the boronic ester‐linked framework. The absorption profile of **NSPh‐Hx** remained largely similar in dry 1,4‐dioxane, tetrahydrofuran (THF), and *N*,*N*‐dimethylformamide (DMF) (Figure 5b)—paralleling the solvent‐independence observed for **TripPh‐Hx** and **Ref‐L** (Figures S8 and S9)—, whereas gradual color changes in DMF indicate partial decomposition over time. Although the lower size limit for dispersible nanosheets was not fully established, these results suggest that the nanosheet assemblies can be dispersed in selected anhydrous organic solvents, although their stability is solvent dependent.

**FIGURE 5:**
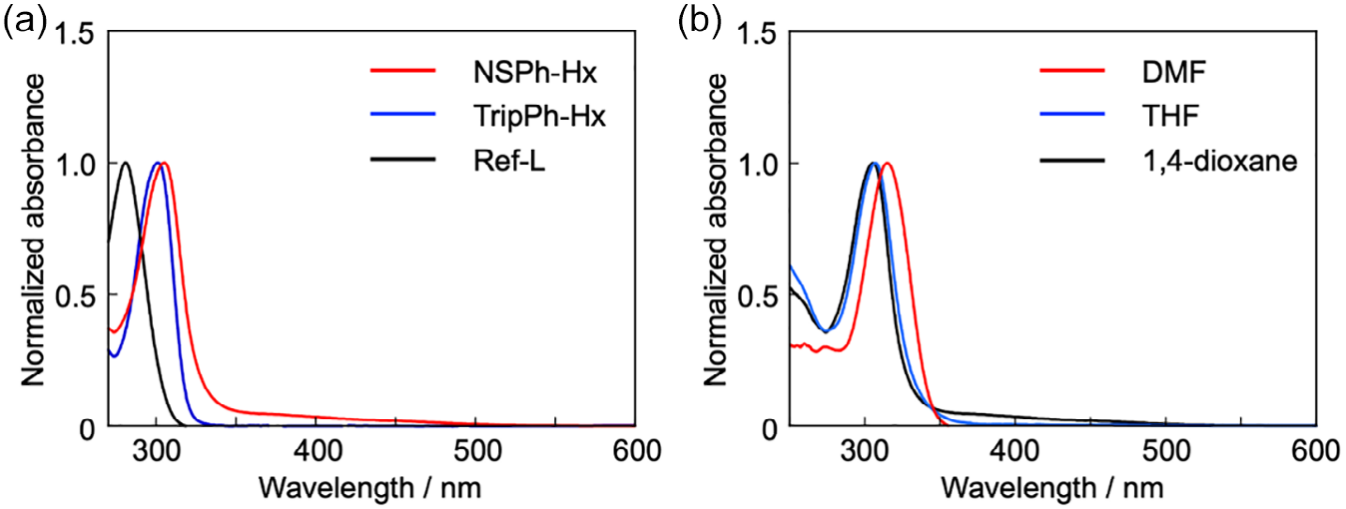
(a) Normalized absorption spectra of **NSPh‐Hx** and reference molecules (**TripPh‐Hx** and **Ref‐L**) in anhydrous 1,4‐dioxane. (b) Normalized absorption spectra of **NSPh‐Hx** in different anhydrous solvents.

Finally, structural diversification through variation of the diboronic acid linker was examined. Such linker variation is important for expanding the structural scope of triptycene‐based nanosheet assemblies. The hexyl‐substituted precursor **1c** was reacted with several aromatic diboronic acids, including 4,4′‐biphenyldiboronic acid, *p*‐terphenyl‐4,4″‐diboronic acid, and pyrene‐2,7‐diboronic acid, affording **NSBP‐Hx**, **NSTP‐Hx**, and **NSPy‐Hx**, respectively. These linkers range from linear oligophenylene units to a polycyclic aromatic structure, providing different rigidity and π‐conjugation lengths. AFM observations after drop‐casting onto mica substrates showed that **NSBP‐Hx**, and **NSPy‐Hx** formed relatively clear nanosheet‐like morphologies (Figure 6). In contrast, the **NSTP‐Hx** showed only partially sheet‐like regions, and aggregated domains were frequently observed near these structures. Although these observations indicate that terphenyl linker can produce sheet‐like features in some regions, the morphology is less uniform and less well‐defined than those obtained with other linkers, at least based on the present AFM observations. This behavior may be related to the lower dispersibility or greater conformational flexibility of the terphenyl‐linked nanosheets, although this interpretation remains tentative. Therefore, the **NSTP‐Hx** is described as a partially sheet‐like assembly rather than a well‐defined nanosheet. In all cases, height profile analysis indicated thicknesses ranging from several nanometers to more than 10 nm, depending on the linker and measured region, consistent with the formation of multilayered sheet‐like assemblies. These results demonstrate that the present synthetic approach potentially accommodates different aromatic diboronic acid linkers and provide access to structurally varied triptycene‐based nanosheet assemblies.

**FIGURE 6:**
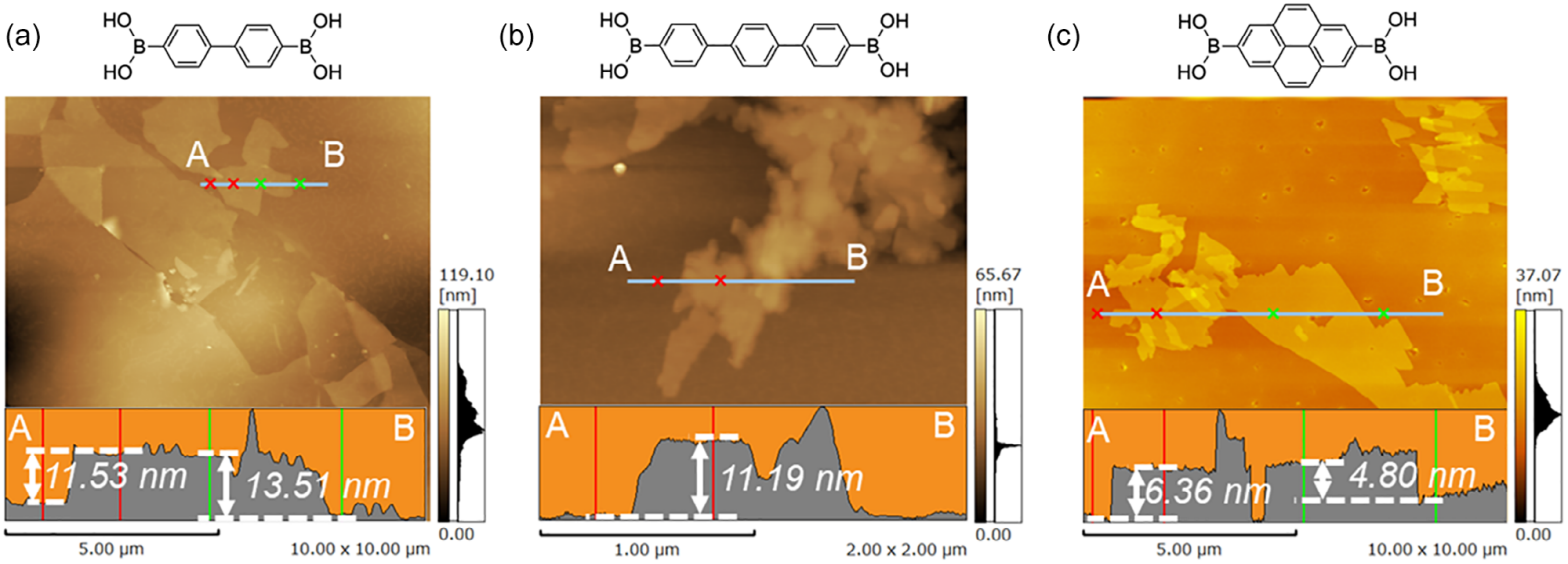
AFM images and height profiles of (a) **NSBP‐Hx**, (b) **NSTP‐Hx**, and (c) **NSPy‐Hx**. Diboronic acids for the corresponding nanosheet formation are also shown.

## 3 Conclusion

In conclusion, triptycene‐based organic nanosheet assemblies were prepared through reversible boronic ester formation between hexahydroxylated triptycene precursors and aromatic diboronic acids. The orthogonal arrangement of alkyl substituents and boronic ester‐forming sites, supported by crystallographic analysis of partial molecular analogs, provides a useful molecular platform for examining substituent effects in triptycene‐based nanosheet assemblies. Time‐dependent microscopy revealed that the alkyl chain length influences the formation and aggregation behavior of these assemblies. Methyl‐substituted precursors showed slower formation of less‐defined and more defective sheet‐like morphologies, whereas hexyl‐substituted precursors produced sheet‐like domains at an earlier stage, followed by aggregation into multilayered assemblies. The recovered mass ratios of the aggregated assemblies also increased with alkyl chain length, providing a comparative indication of alkyl‐chain‐length‐dependent aggregation under the present conditions. Absorption measurements showed a slight red‐shift relative to reference compounds, consistent with modest extension of electronic communication within the boronic ester‐linked framework. Furthermore, variation of the aromatic diboronic acid linkers afforded related sheet‐like assemblies, although the morphology depended on the linker structure and was less well‐defined for the terphenyl‐linked material. Overall, this work provides a case study showing that peripheral alkyl substituents can influence the formation, morphology, and aggregation behavior of triptycene‐based organic nanosheet assemblies. These findings offer useful information for the development of dispersible 2D organic materials based on 3D molecular scaffolds.

## Funding

This study was supported by JST PRESTO (JPMJPR21AC), the Japan Society for the Promotion of Science (JP24K01576, JP26K22862, JP26K01575, JP26H00381, and JP25K01751), the Sumitomo Electric Group Corporate Social Responsibility Foundation, the Hosokawa Powder Technology Foundation, the Inamori Foundation, the Kanamori Foundation, and the Advanced Research Infrastructure for Materials and Nanotechnology (JPMXP1225NM5003 and JPMXP1225NM5140).

## Conflicts of Interest

The authors declare no conflicts of interest.

## Supporting information

Detailed synthetic protocols, single‐crystal X‐ray analysis, optical properties, and details regarding the analytical methods are provided in the Supporting Information. Deposition numbers 2559662 (for **TripPh‐Hx**), 2559664 (for **TripPh‐Me**), and 2559665 (for **Ref‐L**) contain the supplementary crystallographic data for this paper. These data are provided free of charge by the joint Cambridge Crystallographic Data Centre and Fachinformationszentrum Karlsruhe Access Structures service.

## Data Availability

The data that supports the findings of this study are available in the supporting information of this article.
