# Supplementary material for: Triptycene‐Based Organic Nanosheet Assemblies via Reversible Boronic Ester Formation: Alkyl Chain Length‐Dependent Formation and Aggregation
Source: Chempluschem. 2026 Oct 27;91(11):e70276. doi: 10.1002/cplu.70276 (PMC13713105; doi:10.1002/cplu.70276)
Supplement: Supplementary file 1 [file CPLU-91-e70276-s001.pdf]

Supporting Information

Table of Contents

1. Materials and Methods	S2
2. Abbreviations	S2
3. Sample preparation	S2
4. Synthesis	S4
5. X-ray single crystal analysis	S10
6. High-magnification STEM imaging	S14
7. AFM observation of **NSPh-Hx**	S14
8. Photo-image of reaction mixture	S15
9. Proposed assignment of oxidation-related species	S16
10. AFM observation of **TripPh-Hx** and **TripPh-Me**	S17
11. Boronic ester bond dissociation	S18
12. Absorption spectra	S19
13. NMR	S20
14. MS	S25

1. Materials and Methods

1-1. Synthesis and characterization

Reagents for synthesis were purchased from Wako, Nacalai Tesque, and Sigma Aldrich, and were reagent-grade quality, obtained commercially, and used without further purification. For spectral measurements, spectral-grade solvents were purchased from Nacalai Tesque. Unless stated otherwise, column chromatography was carried out on silica gel 60N (Kanto Chemical, 40–60 μm). Analytical thin layer chromatography (TLC) was performed on Art. 5554 (Merck, KGaA). IR spectra were recorded on a JASCO FT/IR-4200 and FT/IR-6700, and are reported as wavenumbers ν in cm^{-1} with band intensities indicated as s (strong), m (medium), w (weak). ^{1}H NMR (400, 500, and 600 MHz) and ^{13}C NMR (100 MHz) spectra were recorded (as indicated) either on a JEOL JNM-ECX400P or JEOL JNM-ECA 600 spectrometer and are reported as chemical shifts (δ) in ppm relative to TMS ($\delta = 0$). Broad peaks are marked as br. High-resolution MS was performed on a Bruker Autoflex II and a Bruker timsTOF APCI-QTOF atmospheric pressure chemical ionization (APCI). The X-ray crystallographic data were recorded at 103 K on a Rigaku R-AXIS RAPID/S using Mo-Kα radiation. UV/Vis absorption spectra were measured using a JASCO UV/Vis/NIR spectrophotometer V-780. AFM observation was performed under ambient conditions using a SPM-9700HT (Shimadzu) in dynamic mode. STEM and SEM observations were conducted using SU-9000 (HITACHI).

2. Abbreviations

AFM: Atomic force microscopy; aq.: Aqueous solution; Ar: Argon; DCM: Dichloromethane; MeOH: Methanol; SEM: Scanning electron microscopy; STEM: Scanning transmission electron microscopy; THF: Tetrahydrofuran; HRMS: High resolution mass spectroscopy; MALDI-TOF: Matrix assisted laser desorption/ionization time-of-flight; rt: room temperature; TLC: Thin layer chromatography.

3. Sample preparation

Microscopic measurements: For STEM and SEM sample preparation, approximately 0.2 ml of the crude reaction mixture, including the precipitated white nanosheet aggregates, was collected and diluted tenfold with dry-toluene. The resulting dispersion was sonicated for 1 min to disperse the aggregates. For STEM observation, one drop of the dispersion was deposited onto a carbon-film-coated TEM grid (UHR-C10). The grid was then dried under vacuum at room temperature for 24 h before STEM and SEM measurements. For SEM observation of the control samples prepared from either the triptycene precursor alone or phenyl 1,4-diboronic acid alone, the dispersion was deposited onto a Si/SiO_2 substrate. The

substrate was dried under vacuum at room temperature for 24 h before SEM measurements. STEM and SEM measurements were carried out at an accelerating voltage of 30 kV with an emission current of 10500 nA. Because the nanosheet assemblies are composed of organic molecular frameworks and are sensitive to electron-beam irradiation, high-magnification STEM imaging was performed carefully, mainly on relatively thick nanosheet regions that could better tolerate electron-beam exposure.

For AFM sample preparation, approximately 0.2 ml of the crude reaction mixture, including the precipitated white nanosheet aggregates, was collected and diluted tenfold with dry-toluene. The resulting dispersion was sonicated for 1 min and the dispersion was drop-cast onto a freshly cleaved mica substrate. The sample was dried under vacuum at room temperature for 24 h before AFM observation. The surface roughness of the freshly cleaved mica substrate was evaluated for nanosheet height analysis. AFM imaging of the bare mica surface gave a root-mean-square roughness of R_q = 0.13 nm, confirming that the substrate roughness was sufficiently smaller than the observed nanosheet heights.

IR measurements: The crude reaction mixture was centrifuged to collect the precipitated nanosheet assemblies. The supernatant was removed by decantation, and dry-dichloromethane or dry-acetonitrile, approximately 5–10 ml, was added to the precipitate. The mixture was sonicated to wash and redisperse the solid, followed by centrifugation to recollect the precipitate. This washing procedure was repeated three times. The obtained solid was dried under vacuum using a Kugelrohr apparatus at 60 °C for at least 2 h, and then used for IR measurements.

4. Synthesis

Hexahydroxylated triptycene derivatives (**1a**–**c**) were synthesized with the modified methods following the previous reports [27–29]. As physical properties are the same as the reported values, ^{1}H NMR spectral data are shown here.

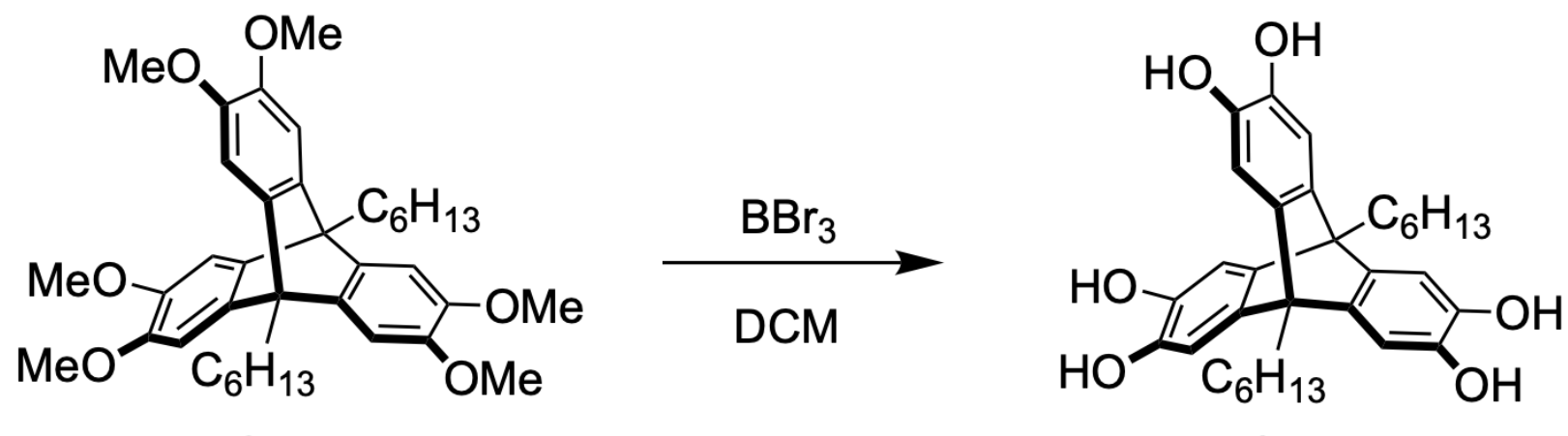

Representative synthetic procedure for precursor

1c (R = C_6H_{13}): To a solution of **2c** (0.2 g, 0.332 mmol) in dry DCM (8 mL) in an ice bath was added dropwise boron bromide (BBr_3) in DCM (1.7 mL, 1 N). After being stirred at rt for 3 h, H_2O was poured into the reaction mixture. The mixture was stirred for another 30 min, and then the resulting precipitate was filtered, washed with water and DCM. The product was then dried under reduced pressure to give **1c** (0.143 g, 83%) as a white solid.

IR (ν cm^{-1}, in KBr): 3371 (s), 2950 (m), 2927 (m), 2852 (w), 1618 (m), 1489 (m), 1446 (s), 1294 (s), 1228 (w), 1205 (w), 1132 (w), 796(w), 775(w), 638(w), 619 (w).; ^{1}H NMR (400 MHz, DMSO-d_6): 8.47 (s, 6H), 6.68 (s, 6H), 2.48–2.44 (t, 4H, J = 7.4 Hz), 1.98–1.91 (m, 4H), 1.76–1.69 (m, 4H), 1.53–1.40 (m, 8H), 1.00–0.97 (t, 6H, J = 7 Hz); ^{13}C NMR (101 MHz, DMSO-d_6): 140.7 (one signal merged), 110.8, 50.8, 32.04, 31.3, 29.09, 25.3, 22.8, 14.6.

1a (R = CH_3). (yield: 93%), ^{1}H NMR (500 MHz, DMSO-d_6): 8.44 (s, 6H), 6.64 (s, 6 H), 2.00 (s, 6H).

1b (R = C_2H_5). (yield: 91%), ^{1}H NMR (500 MHz, DMSO-d_6): 8.42 (s, 6H), 6.70 (s, 6 H), 2.56–2.60 (q, 4H, J = 6.8 Hz), 1.49–1.52 (t, 6H, J = 7.3 Hz).

Synthetic procedure for nanosheets

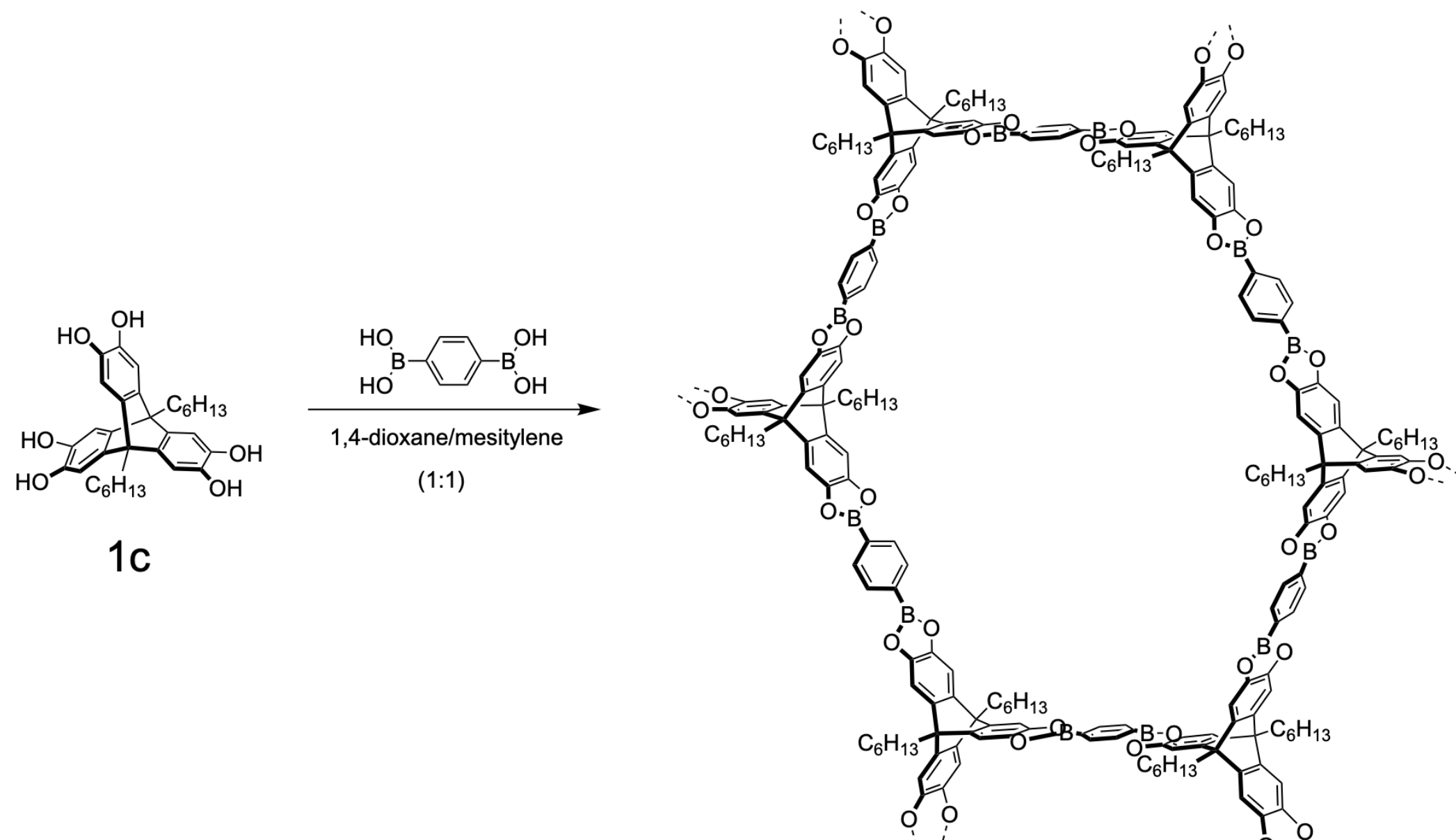

1c

NSPh-Hx

NSPh-Hx (Low concentration conditions): Compound **1c** (5.0 mg, 9.65 μmol, 1 eq) and phenyl 1,4-diboronic acid (2.5 mg, 15.4 μmol, 1.6 eq) were dissolved in dry 1,4-dioxane/mesitylene (40 mL, v/v =1:1) in a 50 mL two-neck flask. The flask was purged with argon and then sealed. The flask was kept at 85 °C for 3 days without disturbance. Afterwards, the tube was allowed to stand and cool to rt, and a white solid **NSPh-Hx** was collected by centrifugation (2.0 mg). IR (ν cm^{-1}, in KBr): 3419 (m), 2952 (m), 2927 (m), 2856 (w), 1608 (m), 1517 (m), 1465 (s), 1396 (s), 1350 (s), 1265 (s), 1079 (m), 1018 (m), 835 (w), 669 (m).

Medium concentration conditions.

Compound **1c** (5 mg, 9.65 μmol, 1 eq) and phenyl 1,4-diboronic acid (2.5 mg, 15.4 μmol, 1.6 eq) were dissolved in dry dioxane/mesitylene (10 mL, v/v =1:1) in a 30 mL two-neck flask. The flask was purged with argon and then sealed. The flask was kept at 85 °C for 3 days without disturbance. Afterwards, the tube was allowed to stand and cool to rt, and a white solid **NSPh-Hx** was collected by centrifugation (6.7 mg). The recovered mass relative to the total mass of the starting materials is calculated to be 0.89.

High concentration conditions.

Compound **1c** (5 mg, 9.65 μmol, 1 eq) and phenyl 1,4-diboronic acid (2.5 mg, 15.4 μmol, 1.6 eq) were dissolved in dry dioxane/mesitylene (2 mL, v/v =1:1) in a 10 mL two-neck flask. The flask was purged with argon and then sealed. The flask was kept at 85 °C for 3 days without disturbance. Afterwards, the tube was allowed to stand and cool to rt, and the obtained black solid was not the target nanosheet.

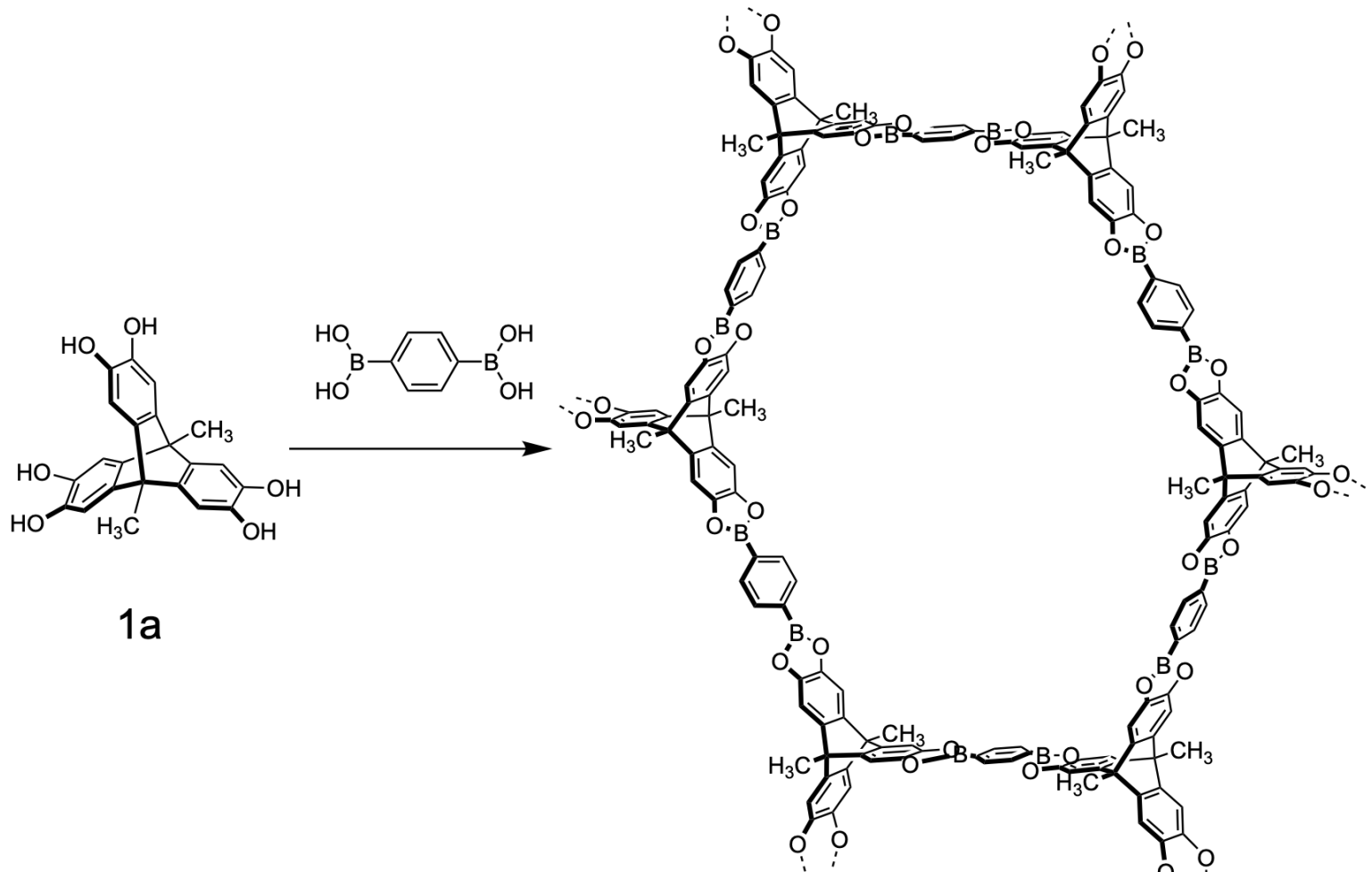

1a

NSPh-Me

NSPh-Me (Low concentration conditions): Compound **1a** (3.6 mg, 9.6 µmol, 1 eq) and phenyl 1,4-diboronic acid (2.5 mg, 15.4 µmol, 1.6 eq) were dissolved in dry 1,4-dioxane/mesitylene (10 mL, v/v =1:1) in a 50 mL two-neck flask. The flask was purged with argon and then sealed. The flask was kept at 85 °C for 3 days without disturbance. Afterwards, the tube was allowed to stand and cool to rt, and a white solid **NSPh-Me** was collected by centrifugation (3.7 mg). The recovered mass relative to the total mass of the starting materials is calculated to be 0.61. IR (ν cm^{-1}, in KBr): 3413 (s), 3297 (s), 2967 (w), 2925 (w), 1624 (m), 1513 (w), 1483 (m), 1452 (m), 1348 (s), 1298 (s), 1126 (s), 1064 (m), 1033 (m), 1010 (w), 877 (w), 814 (w), 667 (w).

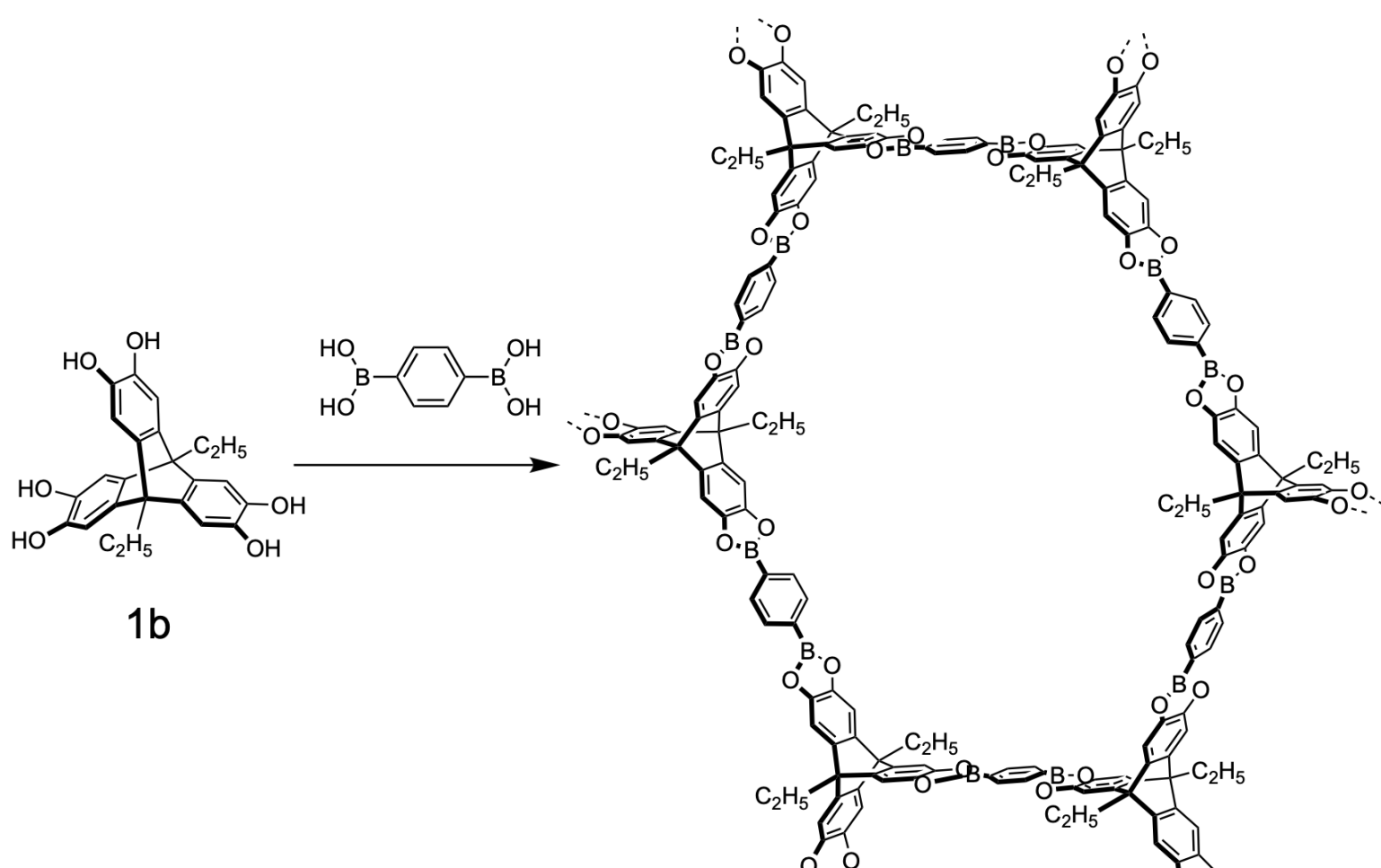

1b

NSPh-Et

NSPh-Et (Low concentration conditions): Compound **1b** (2.0 mg, 4.9 µmol, 1 eq) and phenyl 1,4-diboronic acid (1.3 mg, 7.8 µmol, 1.6 eq) were dissolved in dry 1,4-dioxane/mesitylene (5 mL, v/v =1:1) in a 50 mL two-neck flask. The flask was purged with argon and then sealed. The flask was kept at 85 °C for 3 days without disturbance. Afterwards, the tube was allowed to stand and cool to rt, and a white solid **NSPh-Et** was collected by centrifugation (2.2 mg). The recovered mass relative to the total mass of the starting materials is calculated to be 0.67. IR (ν cm^{-1}, in KBr): 3413 (s), 2921 (w), 1608 (m), 1517 (m),

1467 (m), 1396 (s), 1350 (s), 1263 (m), 1188 (w), 1130 (w), 1080 (m), 1018 (m), 863 (w), 835 (m), 796 (m), 667 (s), 615 (w).

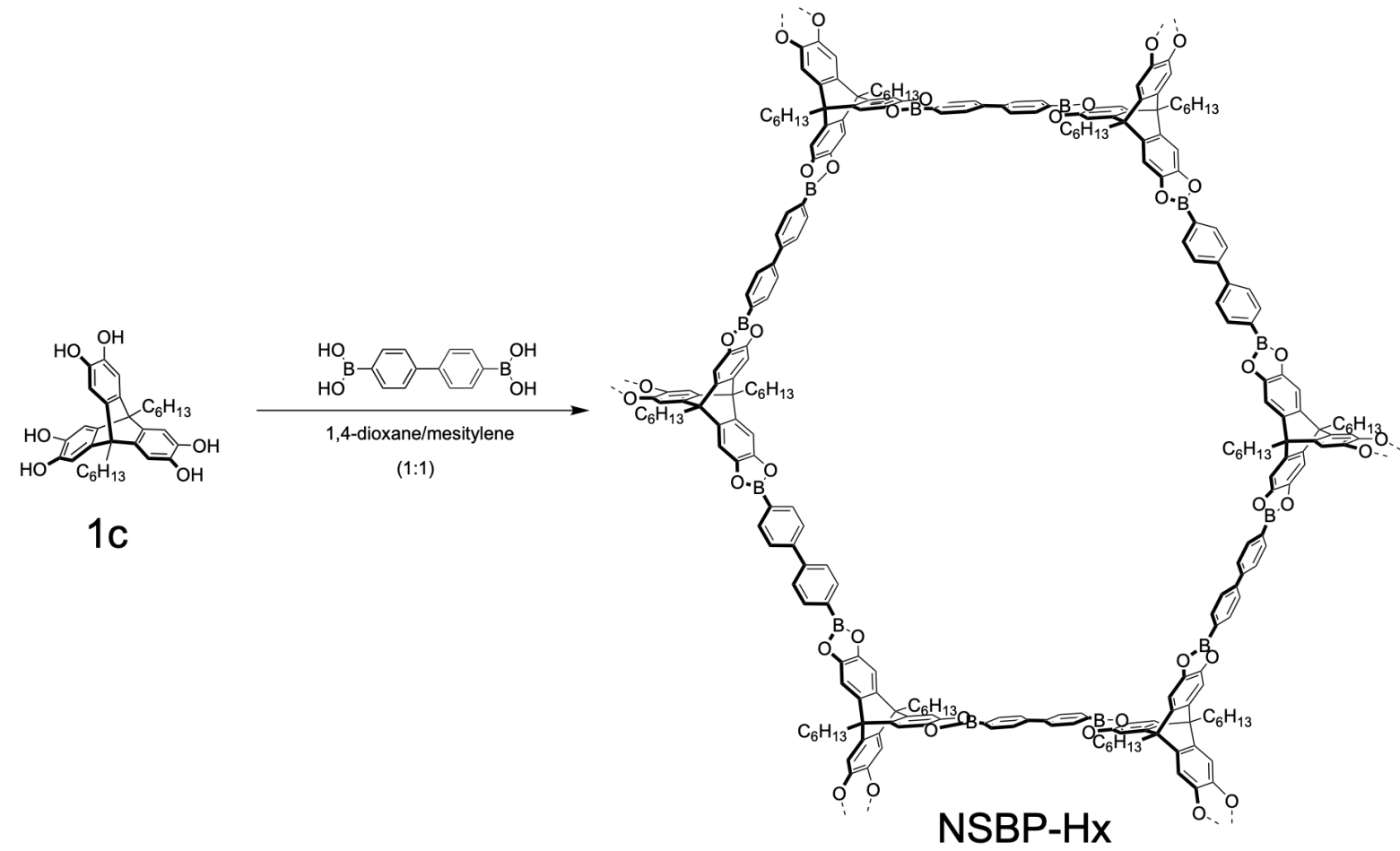

NSBP-Hx: Compound **1c** (10 mg, 19.3 μmol, 1 eq) and 4,4'-biphenyldiboronic acid (7.4 mg, 30.6 μmol, 1.6 eq) were dissolved in dioxane/mesitylene (40 mL, v/v =1:1) in a 50 mL two-neck flask. The flask was purged with argon and then sealed. The flask was kept at 85 °C for 3 days without disturbance. Afterwards, the tube was allowed to stand and cool to rt, and a white solid **NSBP-Hx** was subsequently washed three times with dry acetonitrile, with centrifugation after each wash, and finally collected by filtration (5.0 mg). IR (ν cm^{-1}, in KBr): 3415 (m), 2952 (w), 2927 (w), 2856 (w), 1608 (s), 1465 (m), 1392 (m), 1355 (s), 1267 (m), 1072 (m), 1018 (w), 821 (m), 733 (w), 663 (w).

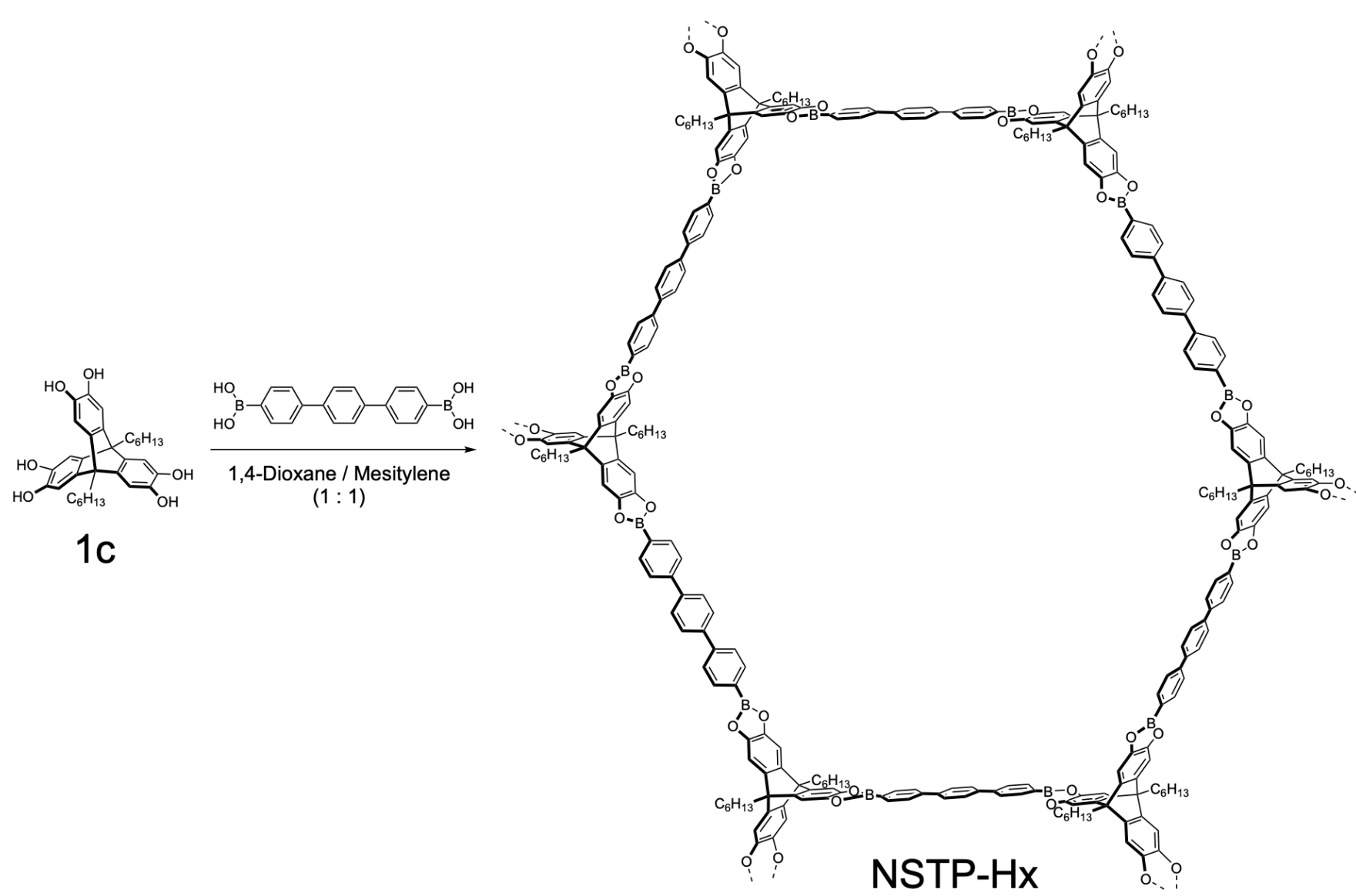

NSTP-Hx: Compound **1c** (5 mg, 9.65 μmol, 1 eq) and *p*-terphenyl-4,4″-diboronic acid (5.2 mg, 16.3 μmol, 1.7 eq) were dissolved in dioxane/mesitylene (20 mL, v/v =1:1) in a 50 mL two-neck flask. The

flask was purged with argon and then sealed. The flask was kept at 85 °C for 3 days without disturbance. Afterwards, the tube was allowed to stand and cool to rt, and a white solid **NSTP-Hx** was subsequently washed three times with dry acetonitrile, with centrifugation after each wash, and finally collected by filtration. IR (ν cm^{-1}, in KBr): 3544 (s), 3213 (s), 2954 (m), 2923 (m), 2865 (m), 1737 (w), 1691 (m), 1677 (m), 1622 (s), 1564 (s), 1514 (w), 1483 (s), 1367 (m), 1261 (w), 1124 (m), 1003 (m), 806 (s), 727 (m), 607 (m).

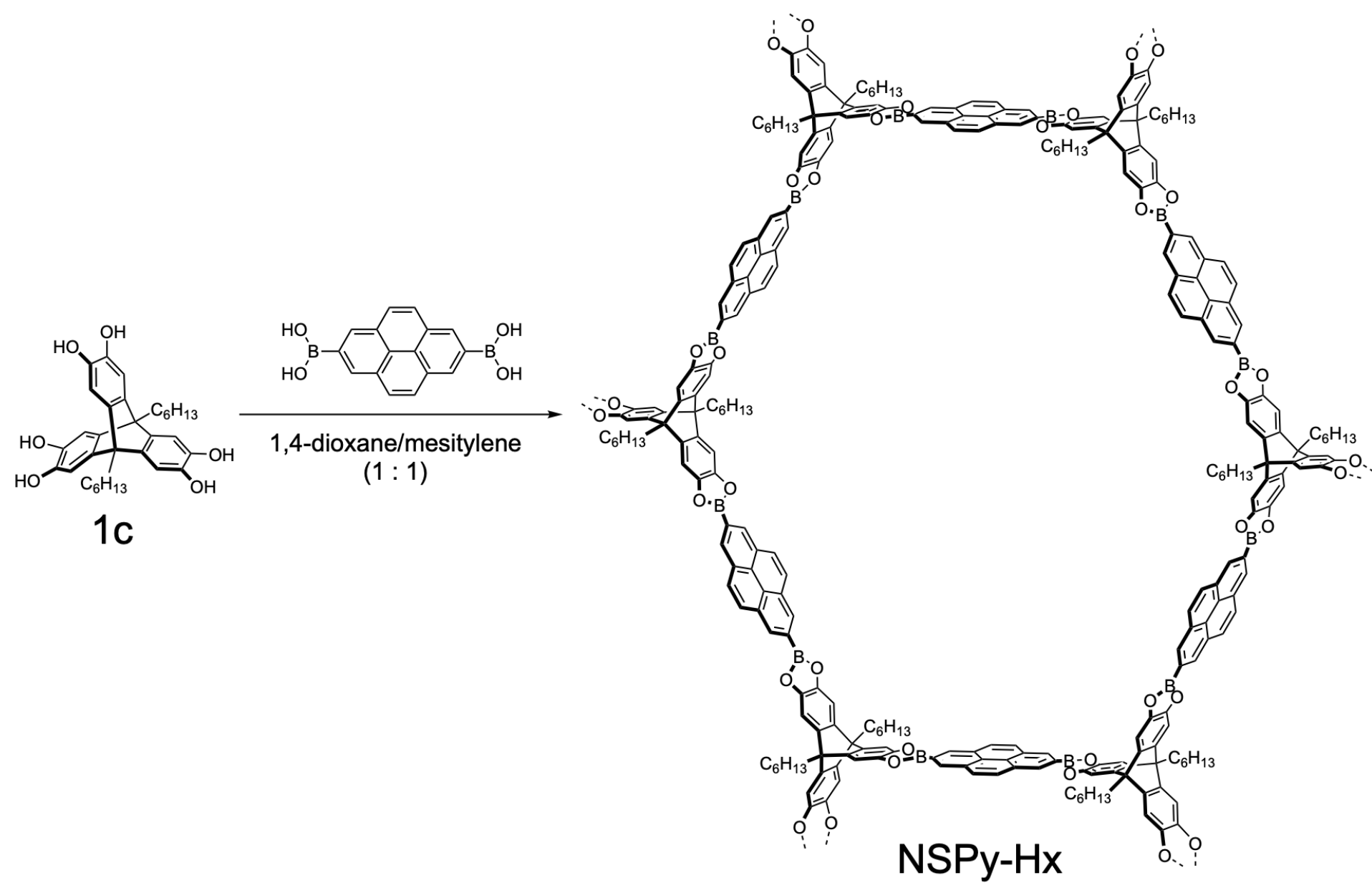

NSPy-Hx: Compound **1c** (2.5 mg, 4.82 μmol, 1 eq) and pyrene-2,7-diyldiboronic acid (2.2 mg, 7.56 μmol, 1.6 eq) were dissolved in dioxane/mesitylene (20 mL, v/v =1:1) in a 50 mL two-neck flask. The flask was purged with argon and then sealed. The flask was kept at 85 °C for 1 day without disturbance. Afterwards, the tube was allowed to stand and cool to rt. A pale-yellow solid was collected by centrifugation (3.1 mg). IR (ν cm^{-1}, in KBr): 3433 (m), 2950 (w), 2925 (w), 2852 (w), 1606 (m), 1460 (s), 1350 (s), 1295 (m), 1230 (s), 895 (w), 798 (w), 719 (m), 682 (w), 669 (w).

Reference compound synthesis

Ref-L was synthesized following the previous report [30].

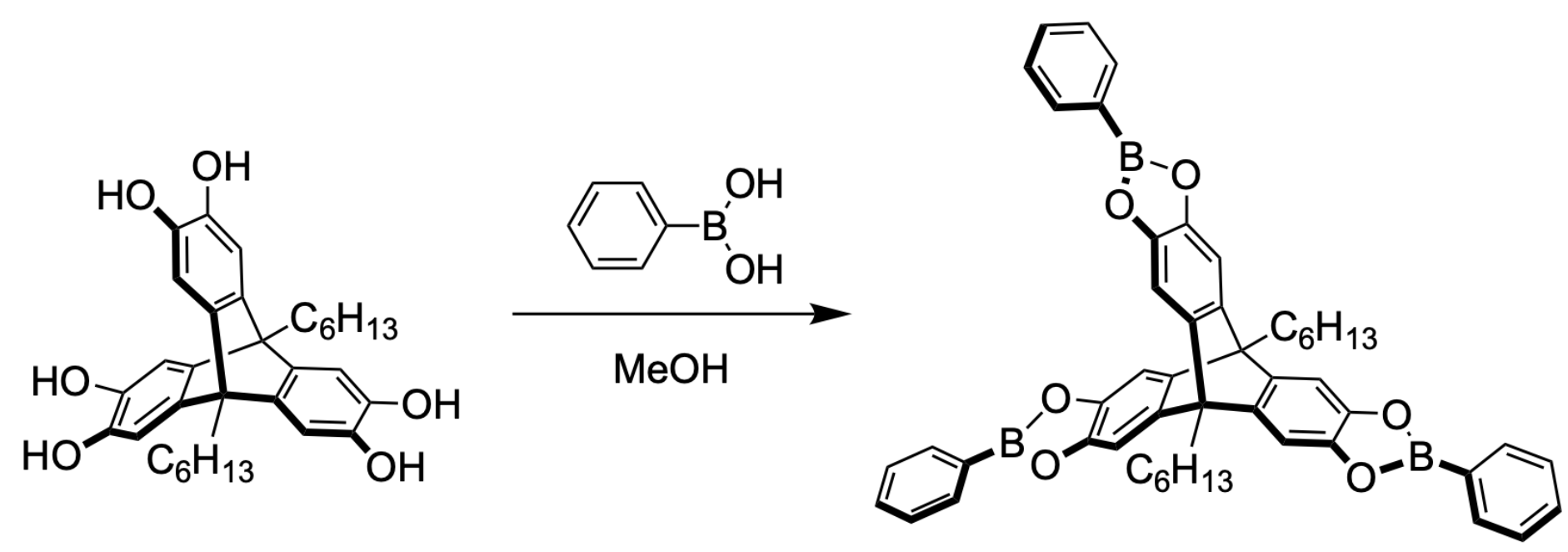

Representative synthetic procedure

TripPh-Hx (R = C_6H_{13}): The compound **1c** (20 mg, 0.0386 mmol, 1 eq) and phenylboronic acid (14.2 mg, 0.116 mmol, 3 eq) were dissolved in methanol (1 mL) and then the solution was evaporated to remove the solvent. The resulting residue was quickly heated to 90 ºC under vacuum for several hours. The residue was reprecipitated with DCM and acetonitrile to give **TripPh-Hx** (21 mg, 69%) as a white solid.

IR (ν cm^{-1}, in KBr): 3438 (w), 3055 (w), 2952 (m), 2925 (m), 2861 (m), 1608 (m), 1462 (s), 1359 (s), 1267 (s), 1184 (m), 1070 (m), 1024 (w), 865 (w), 800 (m), 698 (m), 663 (m), 605 (w); ^{1}H NMR (500 MHz, $CDCl_3$): 8.01–8.03 (d, 6H, *J* = 7.5 Hz), 7.44–7.56 (m, 15H), 2.93–2.96 (t, 4H, *J* = 7.5 Hz), 2.25 (s, 4H), 1.88–1.94(m, 4H), 1.49–1.65 (m, 8H), 1.05–1.08 (t, 6H, *J* = 7.3 Hz); ^{13}C NMR (101 MHz, $CDCl_3$): 134.9, 132.4, 128.3, 53.2, 32.1, 31.6, 29.6, 25.4, 22.9, 14.3.

TripPh-Me (R = CH_3): (57%); IR (ν cm^{-1}, in KBr): 3419 (w), 3076 (w), 3056 (w), 2970 (w), 1606 (m), 1502 (w), 1473 (m), 1452 (s), 1396 (s), 1357 (s), 1313 (w), 1274 (s), 1247 (m), 1186 (m), 1155 (m), 1070 (s), 1024 (m), 875 (m), 773 (m), 698 (s), 665 (m); ^{1}H NMR (500 MHz, $CDCl_3$): 8.01–8.02 (d, 6H, *J* = 6.5 Hz), 7.53–7.56 (t, 3H, *J* = 7.5 Hz), 7.44–7.47 (t, 6H, *J* = 7.3 Hz), 7.38 (s, 6H), 2.51 (s, 6H); ^{13}C NMR (101 MHz, $CDCl_3$): 145.4, 144.01, 134.8, 132.3, 128.2, 106.2, 48.5, 14.9.

5. X-ray single crystal analysis

TripPh-Hx (CCDC: 2559662)

Empirical formula	$C_{50}H_{47}O_6B_3$
Formula weight	776.3
Temperature	103(2) K
Wavelength	0.71075 Å
Crystal system	Triclinic
Space group	*P*-1
Unit cell dimensions	a = 8.6245(2) Å
	b = 11.7133(3) Å
	c = 20.8097(4) Å
	α = 97.934(7) °
	β = 96.503(7) °
	γ = 93.309(7) °
Volume	2062.95(9) $Å^3$
Z	2
Density (calculated)	1.250 g/cm^3
Absorption coefficient	0.079 mm^{-1}
F(000)	820
Crystal size	0.160 × 0.090 × 0.070 mm^3
Theta range for data collection	2.458 to 27.483°
Index ranges	$-11 \leq h \leq 11$, $-15 \leq k \leq 15$, $-27 \leq l \leq 27$
Reflections collected	35071
Independent reflections	9444 [R(int) = 0.0165]
Completeness to theta = 24.495°	99.9%
Max. and min. transmission	1.000 and 0.8681
Refinement method	Full-matrix least-squares on F^2
Data / restraints / parameters	9444 / 0 / 534
Goodness-of-fit on F^2	1.004
Final R indices [$I > 2\sigma(I)$]	R_1 = 0.0414, wR_2 = 0.1106
R indices (all data)	R_1 = 0.0477, wR_2 = 0.1156
Extinction coefficient	n/a
Largest diff. peak and hole	0.549 and –0.167 e.$Å^{-3}$

TripPh-Me (CCDC: 2559664)

Empirical formula	$C_{40}H_{27}O_6B_3 \bullet CH_2Cl_2$
Formula weight	720.97
Temperature	103(2) K
Wavelength	0.71075 Å
Crystal system	Tetragonal
Space group	$I4_1/a$
Unit cell dimensions	a = 32.8953(18) Å
	c = 13.1749(4) Å
Volume	14256.6(16) Å^3
Z	16
Density (calculated)	1.344 g/cm^3
Absorption coefficient	0.231 mm^{-1}
F(000)	5952
Crystal size	0.160 × 0.040 × 0.020 mm^3
Theta range for data collection	1.751 to 24.997°
Index ranges	$-39 \le h \le 39$, $-39 \le k \le 39$, $-15 \le l \le 15$
Reflections collected	93144
Independent reflections	6272 [R(int) = 0.1505]
Completeness to theta = 63.500°	99.8%
Refinement method	Full-matrix least-squares on F^2
Data / restraints / parameters	6272 / 31 / 499
Goodness-of-fit on F^2	1.036
Final R indices [$I > 2\sigma(I)$]	R_1 = 0.0815, wR_2 = 0.2077
R indices (all data)	R_1 = 0.1282, wR_2 = 0.2358
Extinction coefficient	n/a
Largest diff. peak and hole	0.701 and –0.874 e.Å^{-3}

Ref-L (CCDC: 2559665)

Empirical formula	$C_{18}H_{12}O_4B_2$
Formula weight	313.90
Temperature	103(2) K
Wavelength	0.71075 Å
Crystal system	Monoclinic
Space group	*C*2/c
Unit cell dimensions	*a* = 11.2629(6) Å
	b = 5.5839(3) Å
	c = 22.8282(12) Å
	β = 95.665(7) °
Volume	1428.68(13) $Å^3$
Z	4
Density (calculated)	1.459 g/cm^3
Absorption coefficient	0.100 mm^{-1}
F(000)	648
Crystal size	0.090 × 0.040 × 0.020 mm^3
Theta range for data collection	1.793 to 25.351°
Index ranges	$-13 \le h \le 13$, $0 \le k \le 6$, $0 \le l \le 27$
Reflections collected	9688
Independent reflections	1059 [*R*(int) = 0.0685]
Refinement method	Full-matrix least-squares on F^2
Data / restraints / parameters	1321 / 0 / 109
Goodness-of-fit on F^2	1.102
R indices (all data)	R_1 = 0.0826, wR_2 = 0.2153
Extinction coefficient	n/a
Largest diff. peak and hole	0.70 and –0.28 e.$Å^{-3}$

X-ray single crystal structure of TripPh-Me

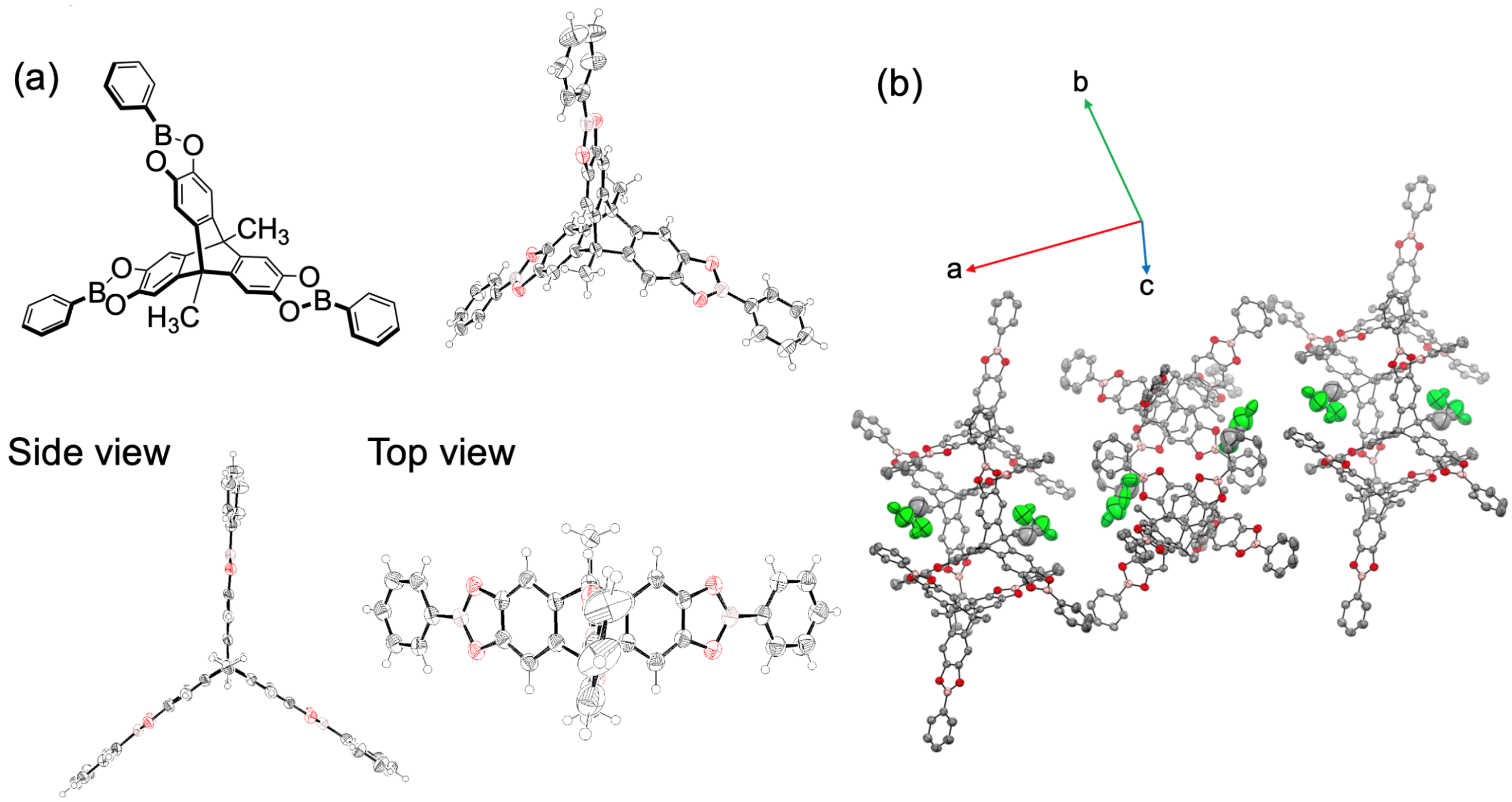

Figure S1. (a) X-ray single crystal structure of **TripPh-Me**, and (b) packing structure. Thermal ellipsoids represent 50% probability. Hydrogen atoms are omitted for clarity for (b).

6. High-magnification STEM imaging

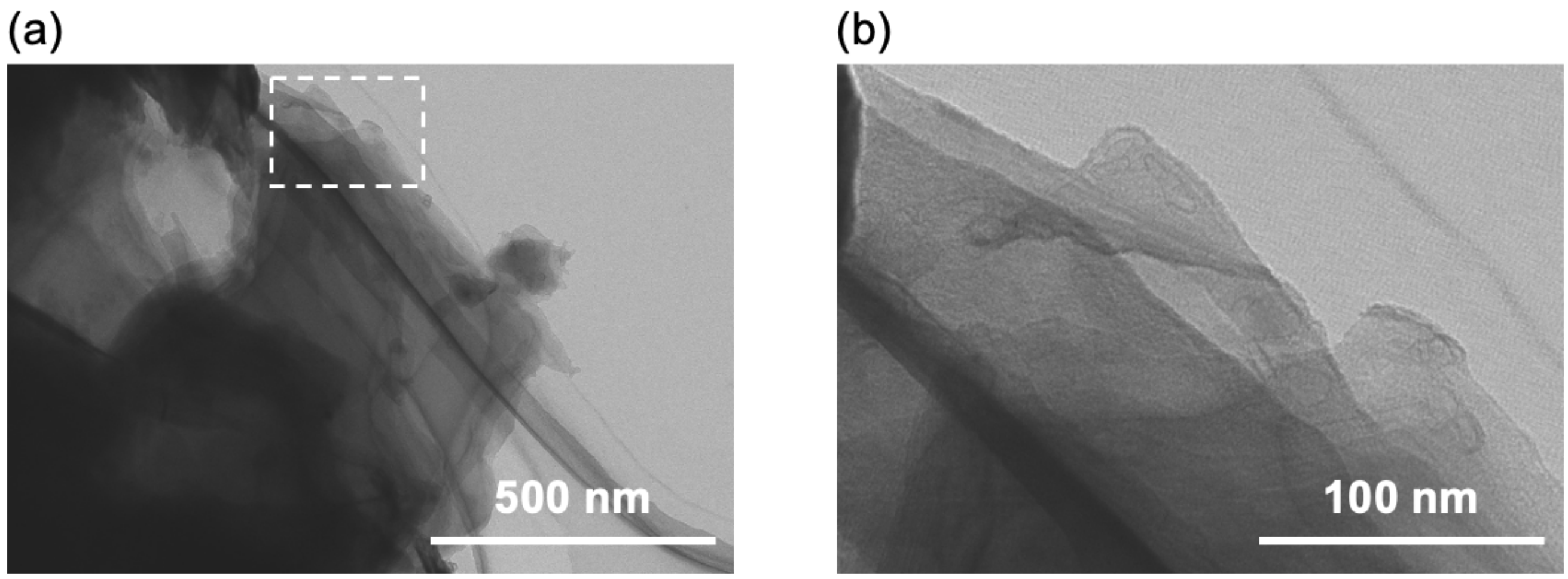

Figure S2. (a,b) STEM images of **NSPh-Hx**. (b) High-magnification STEM image of the area marked by the white dashed square in (a). Relatively thicker regions were selected for magnified observation because thinner organic nanosheet regions were sensitive to electron-beam irradiation.

7. AFM observation of NSPh-Hx

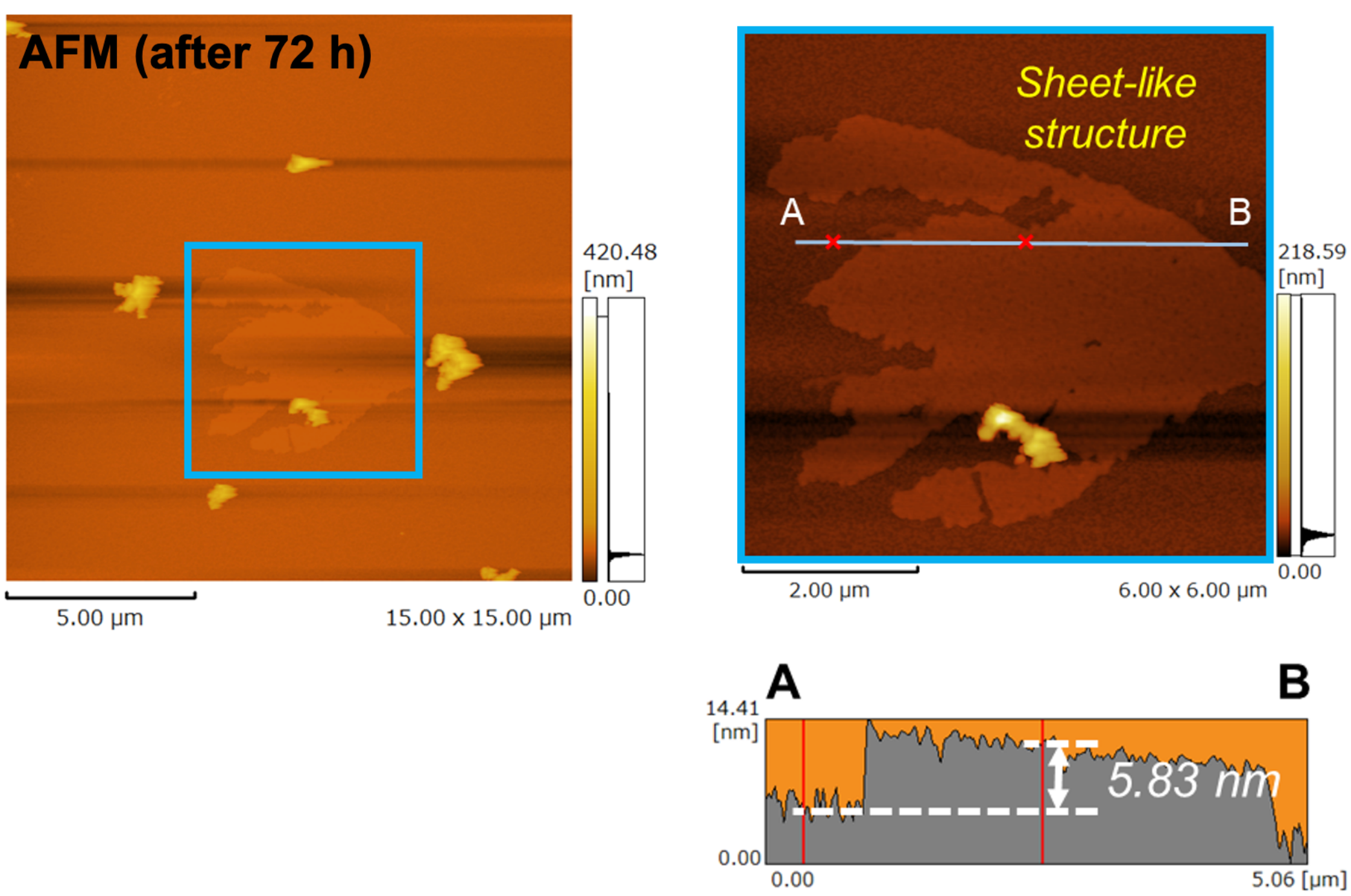

Figure S3. AFM image and height profile of **NSPh-Hx** after 72-hours reaction.

8. Photo-image of reaction mixture

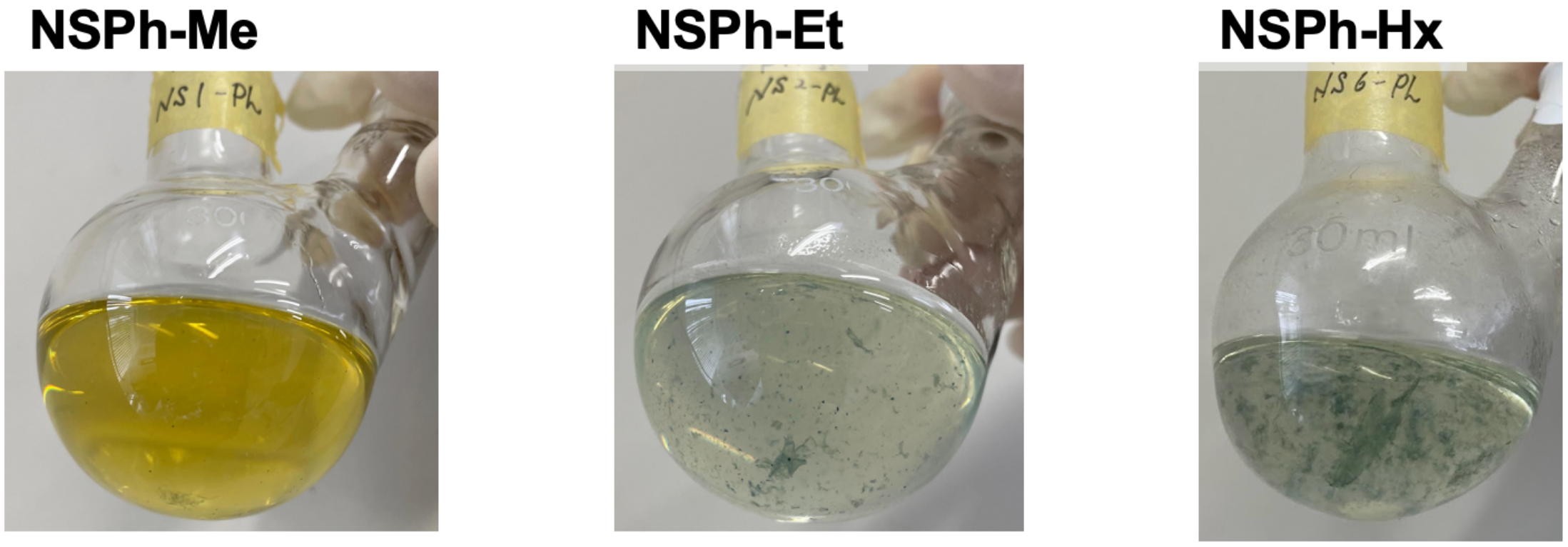

Figure S4. Photo-images of reaction mixtures after 72-hours reaction.

9. Proposed assignment of oxidation-related species

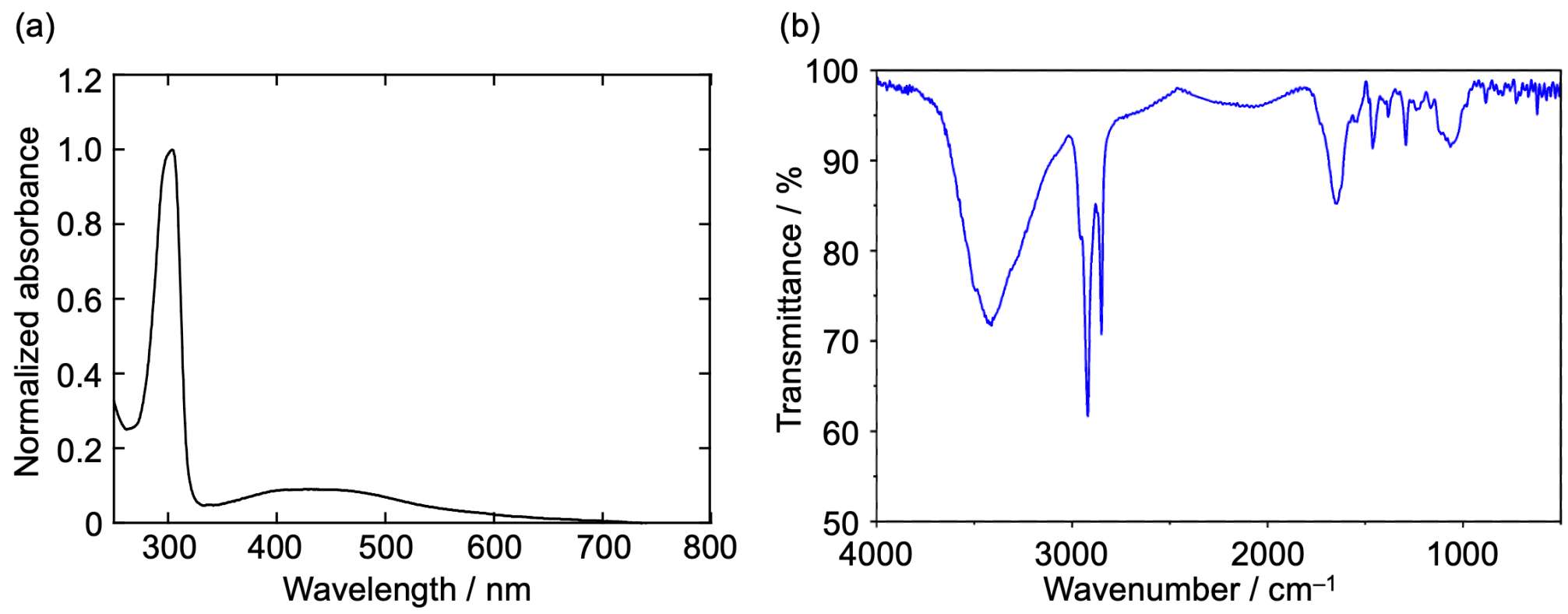

Figure S5. (a) Normalized UV-vis absorption spectrum of the yellow-colored material in acetonitrile and (b) its IR spectrum recorded using the KBr method.

Preparation and characterization of the yellow-colored material: Compound **1a** (4.0 mg) was dissolved in a mixture of 1,4-dioxane/mesitylene (10 ml, 1:1, v/v), and the resulting solution was heated at 85 ºC for 3 days. The solution gradually changed from colorless to yellow. After removal of the solvent, the residue was dried under vacuum at 60 ºC for 2 h. The obtained material was then used as the "decomposed material" for UV/vis absorption, IR, and NMR measurements (Figure S18, S19).

Proposed assignment of the decomposed material: The UV-vis absorption spectrum of the decomposed material showed a characteristic absorption band in the 400–500 nm region. Similar optical features have been reported for related triptycene quinone/quinol compounds [31]. Note that the crude solution for nanosheet formation exhibited the same broad absorption in the 400–500 nm region. Additionally, the ^{1}H NMR spectrum showed signals with chemical shifts similar to those reported for related oxidized triptycene derivatives [31]. The ^{13}C NMR spectrum and IR analysis also indicated the presence of carbonyl groups. These spectroscopic features are consistent with partial oxidation of the catechol-like hydroxy groups in the triptycene-hexaol precursor to quinone-like structures. Because oxidation of the hydroxy groups would reduce the number of sites available for boronic ester formation, such oxidized species are expected to remain in the reaction mixture and may contribute to the yellow coloration. Therefore, the yellow-colored material is tentatively assigned as a mixture of partially oxidized triptycene derivatives containing quinone-like structures.

10. AFM observation of TripPh-Hx and TripPh-Me

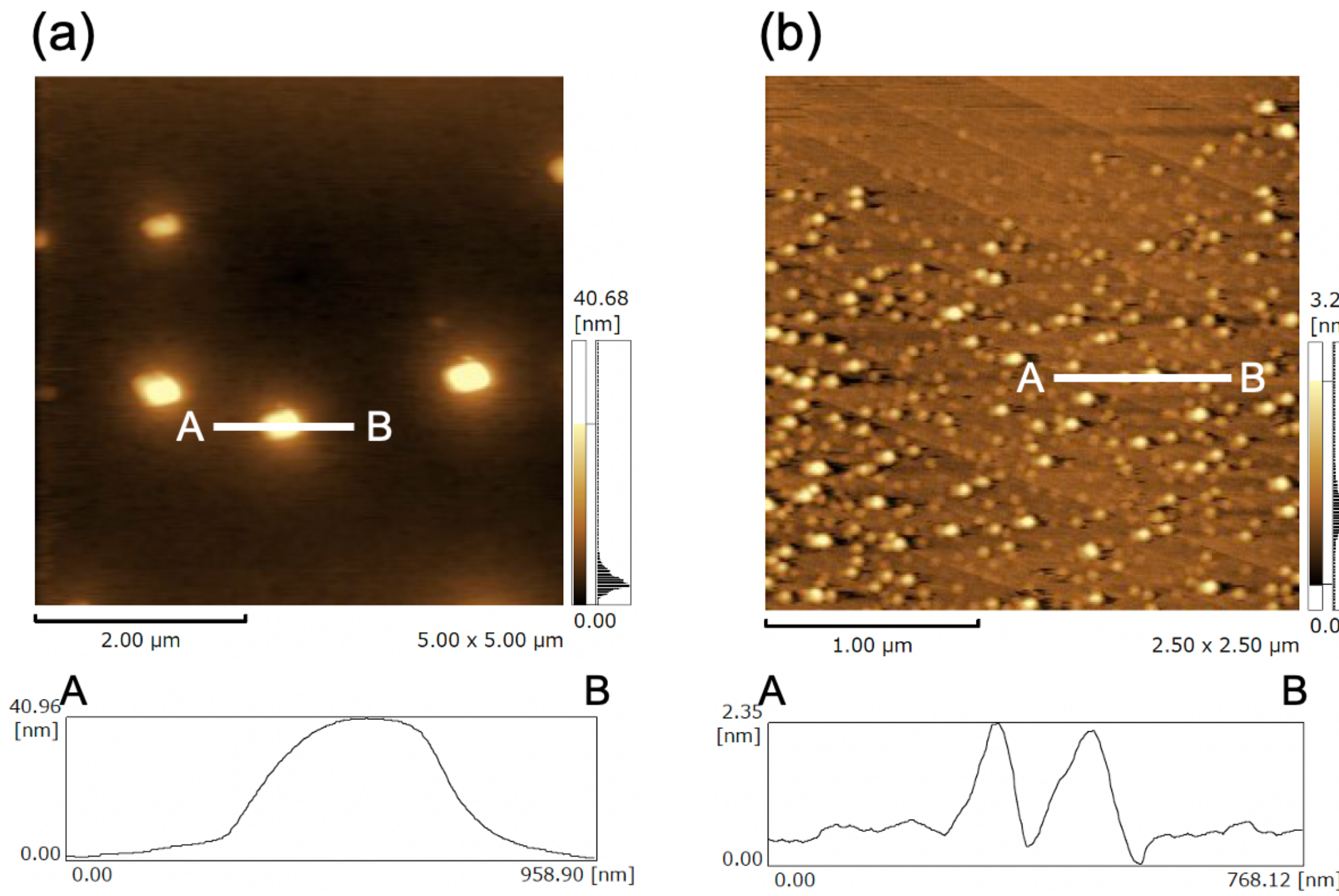

Figure S6. AFM image and height profile of granular aggregated domains for (a) **TripPh-Hx** and (b) **TripPh-Me**.

AFM observation of **TripPh-Hx** mainly showed granular aggregated domains with heights of ~30–50 nm. In contrast, **TripPh-Me** showed relatively featureless thin-film-like regions together with smaller aggregates (heights of ~2–5 nm). These results suggest that hexyl-substituted molecular fragment may have a stronger tendency toward aggregation than the methyl-substituted analogue under comparable deposition conditions. However, because the solubility, dispersibility, and molecular connectivity of the small-molecule fragments differ substantially from those of the extended nanosheet assemblies, we do not claim that these observations directly prove the nanosheet formation mechanism. We present them as supplementary evidence supporting an alkyl-substituent-dependent aggregation tendency at the molecular-fragment level.

Sample preparation: **TripPh-Hx** (0.54 mg) or **TripPh-Me** (0.51 mg) was dissolved in mesitylene/1,4-dioxane (1:1, v/v) (4.0 mL for **TripPh-Hx** and 5.5 mL for **TripPh-Me**). After sonication for 1 min, one drop of the solution was deposited onto freshly cleaved mica. The sample was dried under vacuum at room temperature for 24 h and then used for AFM measurements.

11. Boronic ester bond dissociation

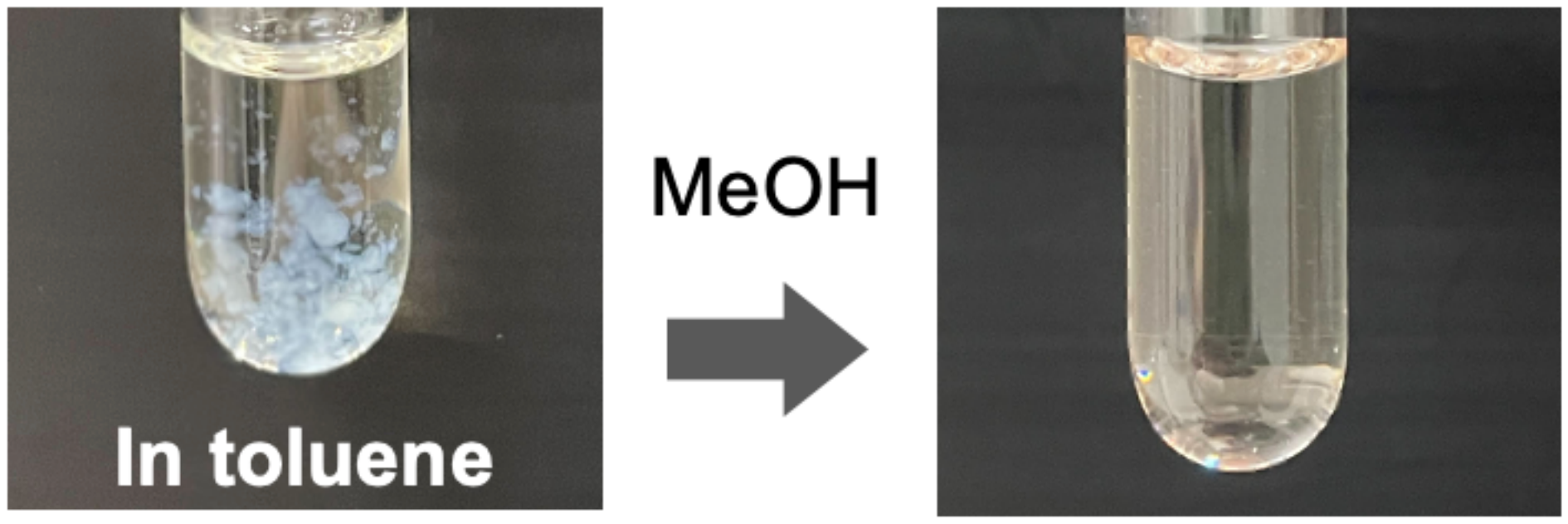

Figure S7. Photographs of the **NSPh-Hx** dispersion in toluene before (left) and after (right) the addition of methanol, showing the disappearance of visible aggregates.

12. Absorption spectra

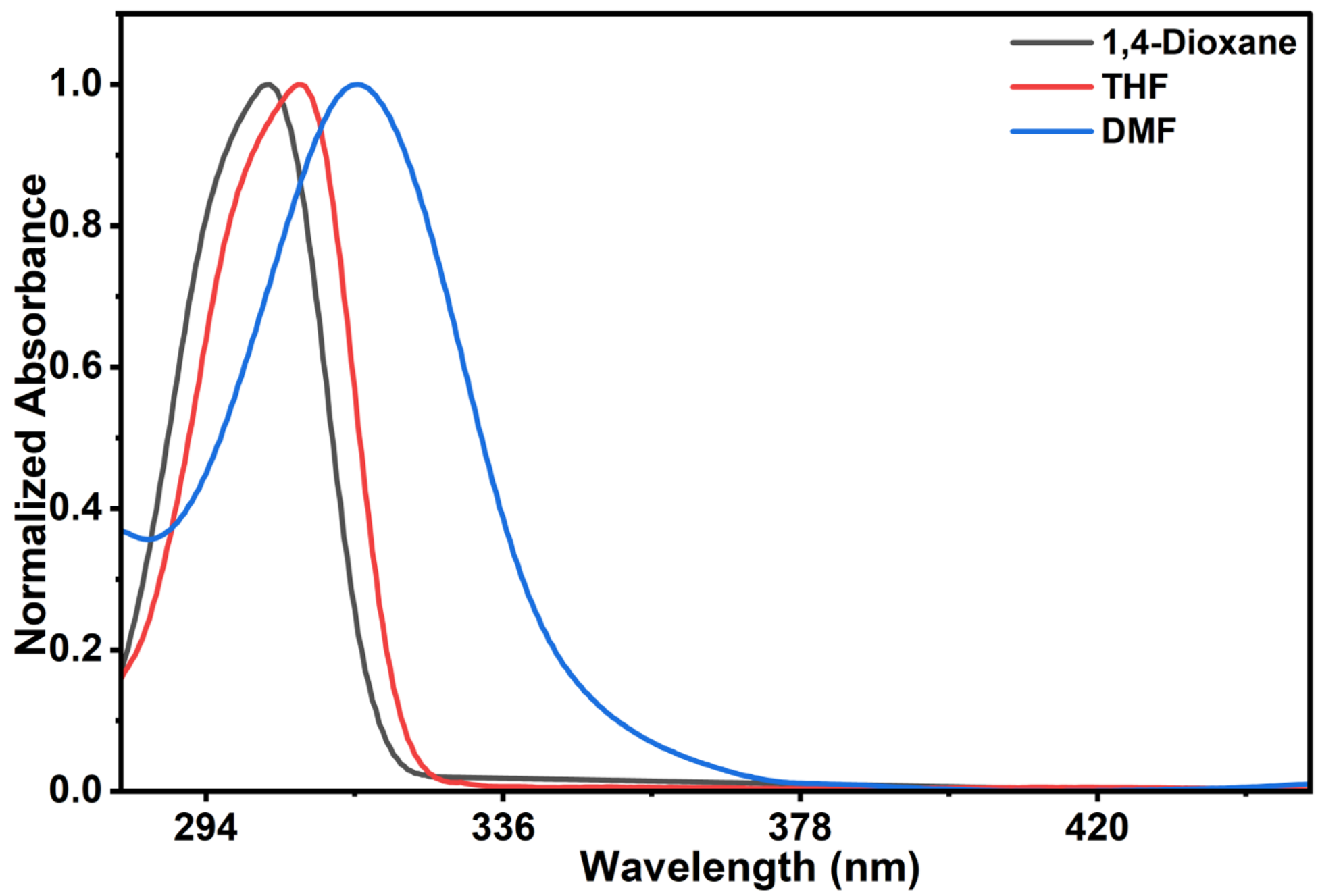

Figure S8. Normalized absorption spectra of **TripPh-Hx** in different solvents.

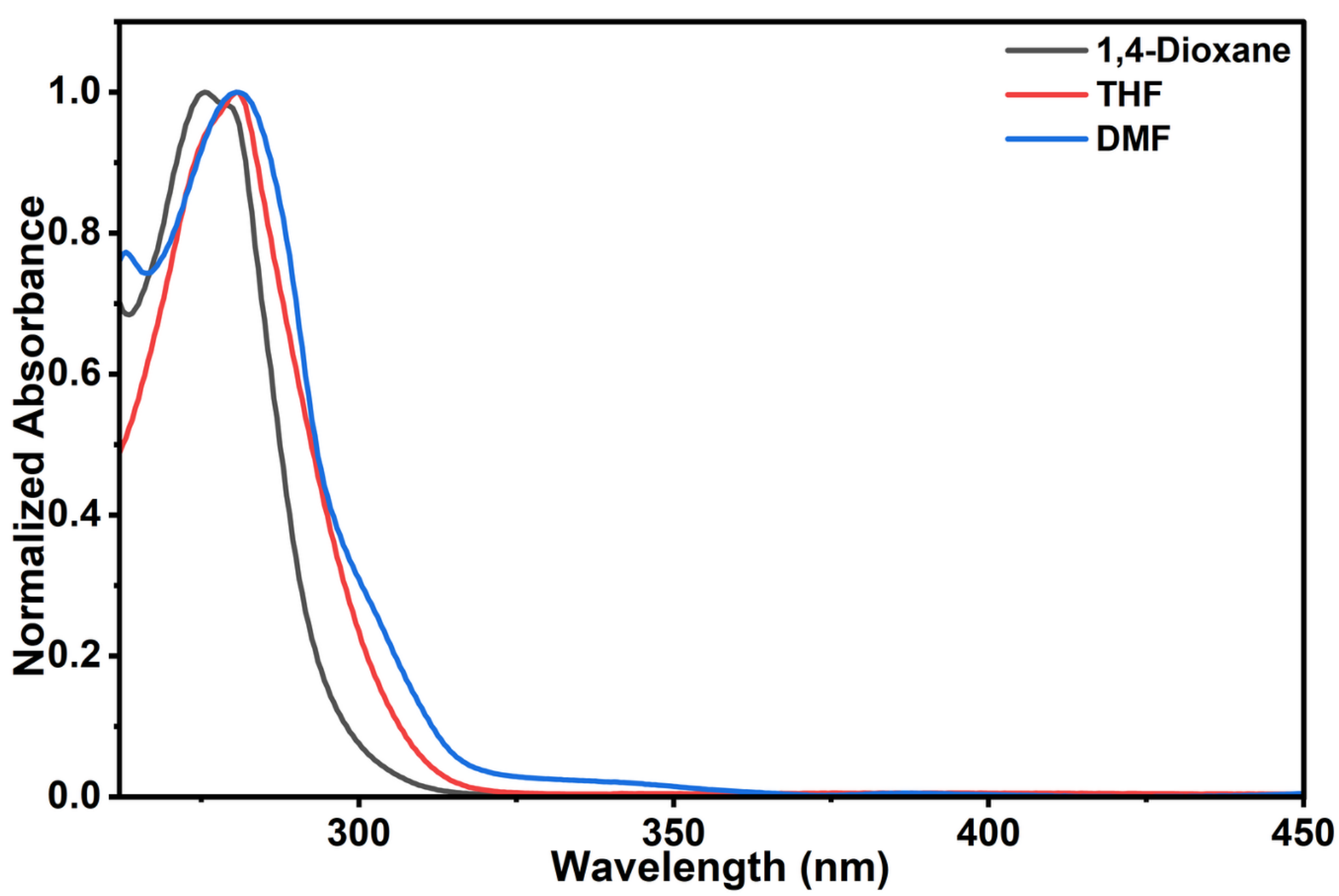

Figure S9. Normalized absorption spectra of **Ref-L** in different solvents.

13. NMR

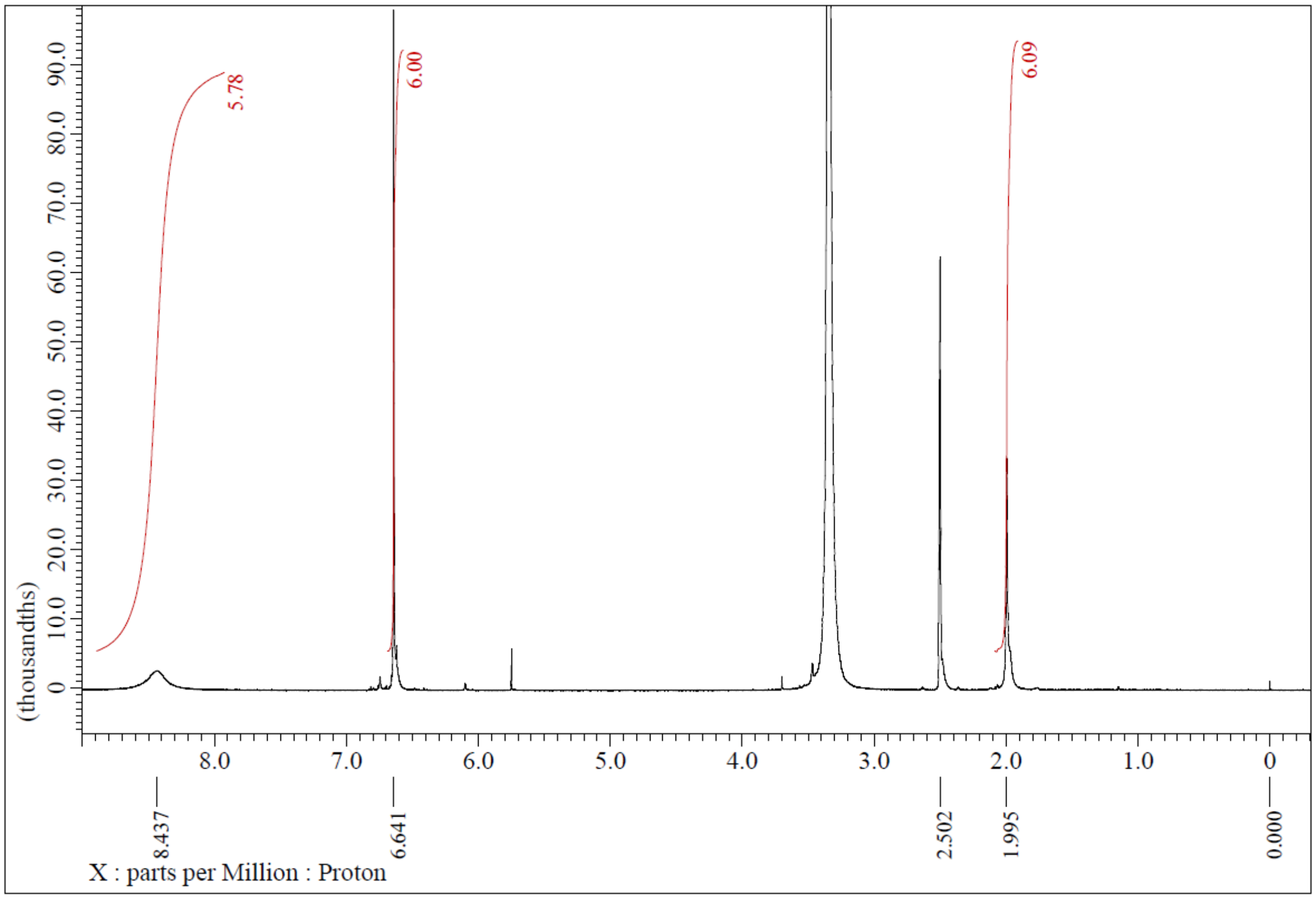

Figure S10. ^{1}H NMR (500 MHz, DMSO-d_6) spectrum of compound **1a**.

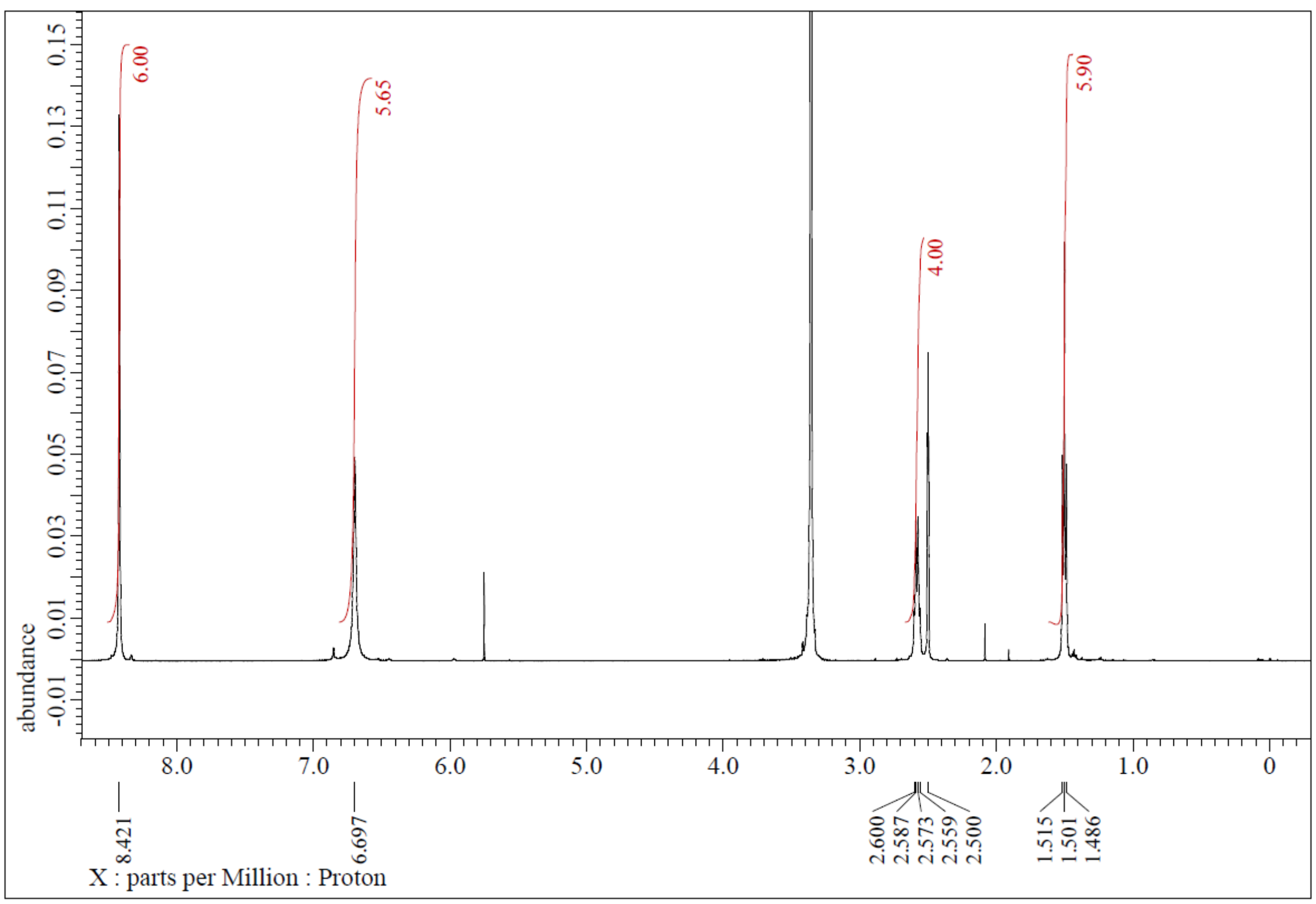

Figure S11. ^{1}H NMR (500 MHz, DMSO-d_6) spectrum of compound **1b.**

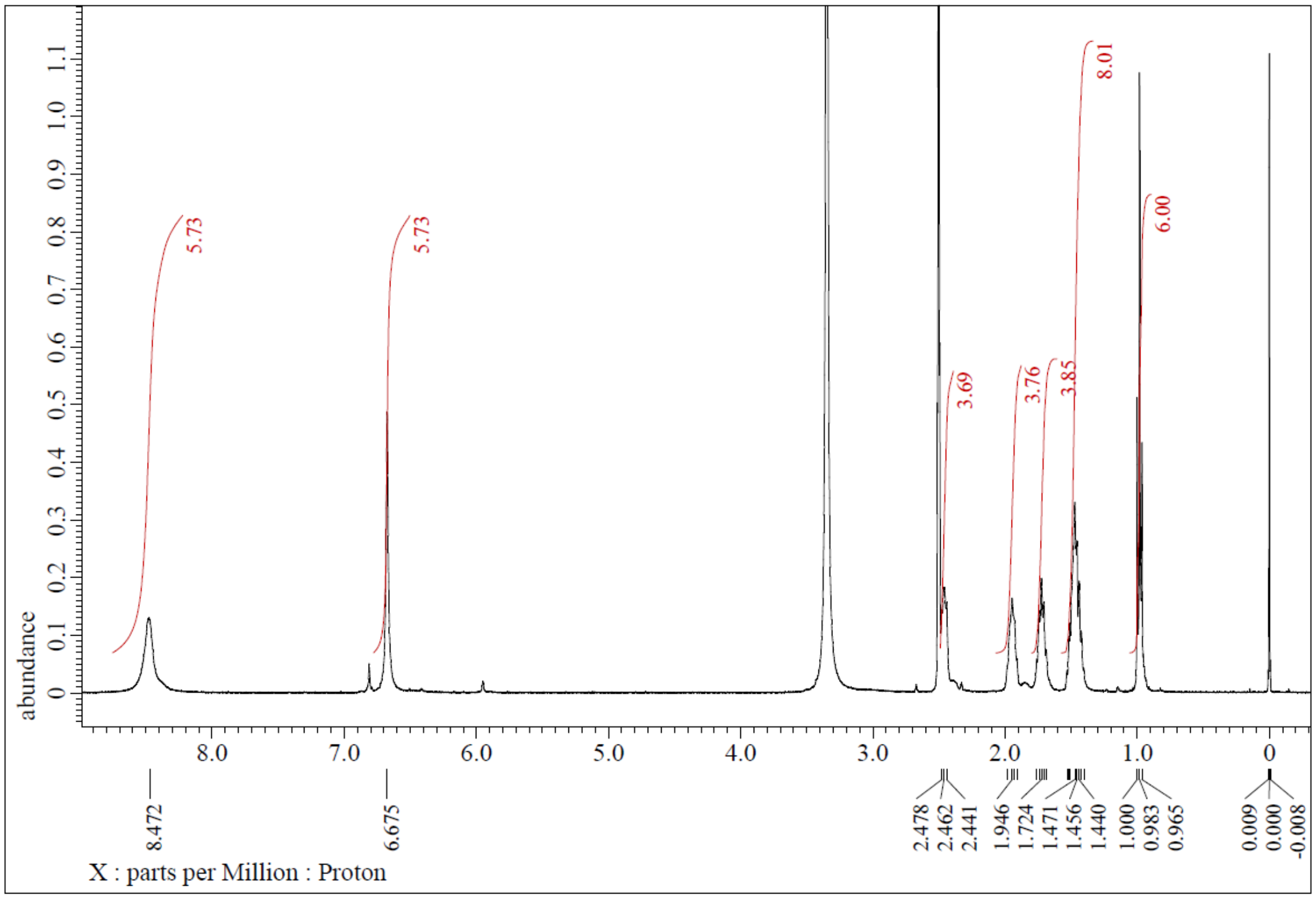

Figure S12. ^{1}H NMR (400 MHz, DMSO-d_6) spectrum of **1c**.

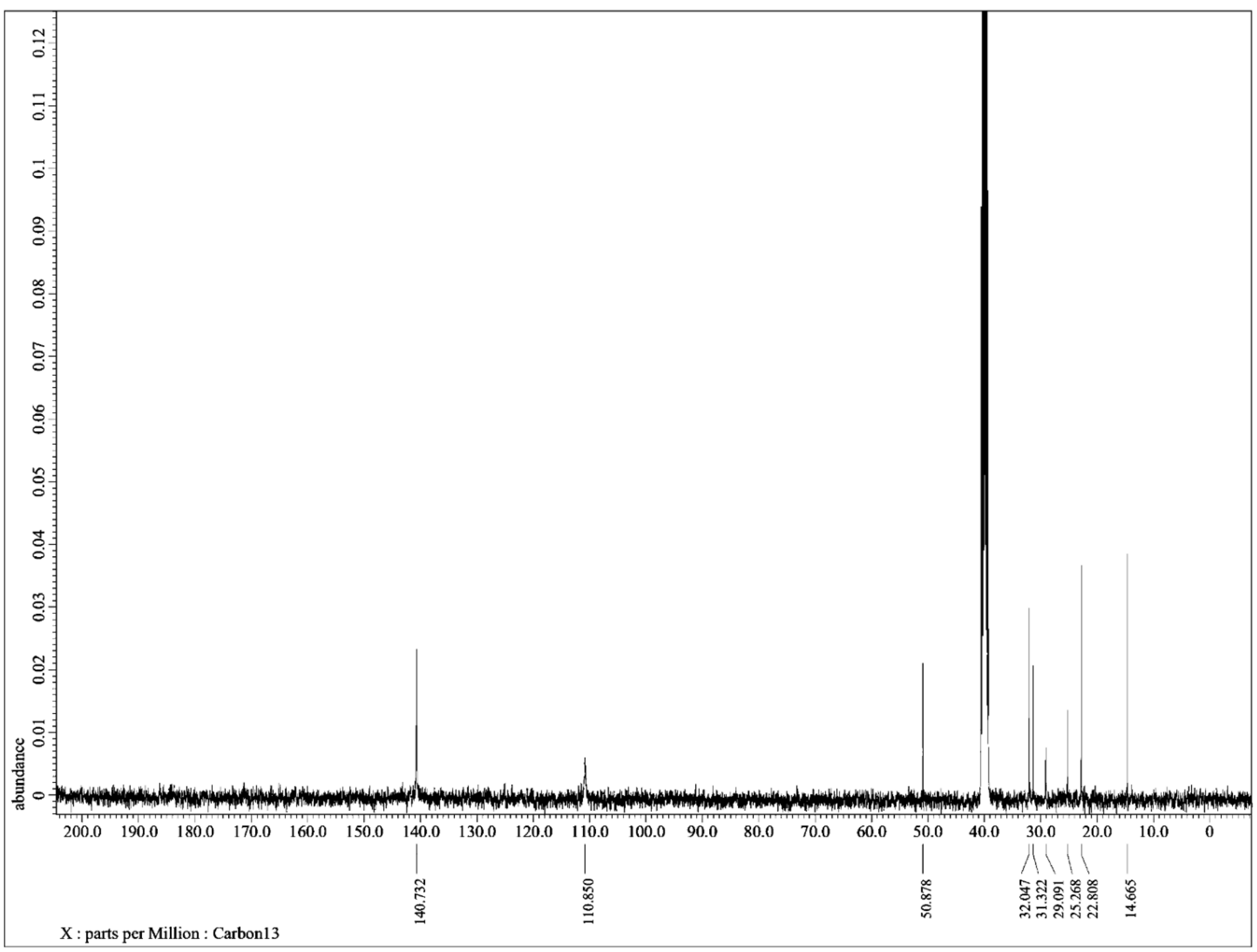

Figure S13. ^{13}C NMR (100 MHz) spectrum of **1c** in DMSO-d_6.

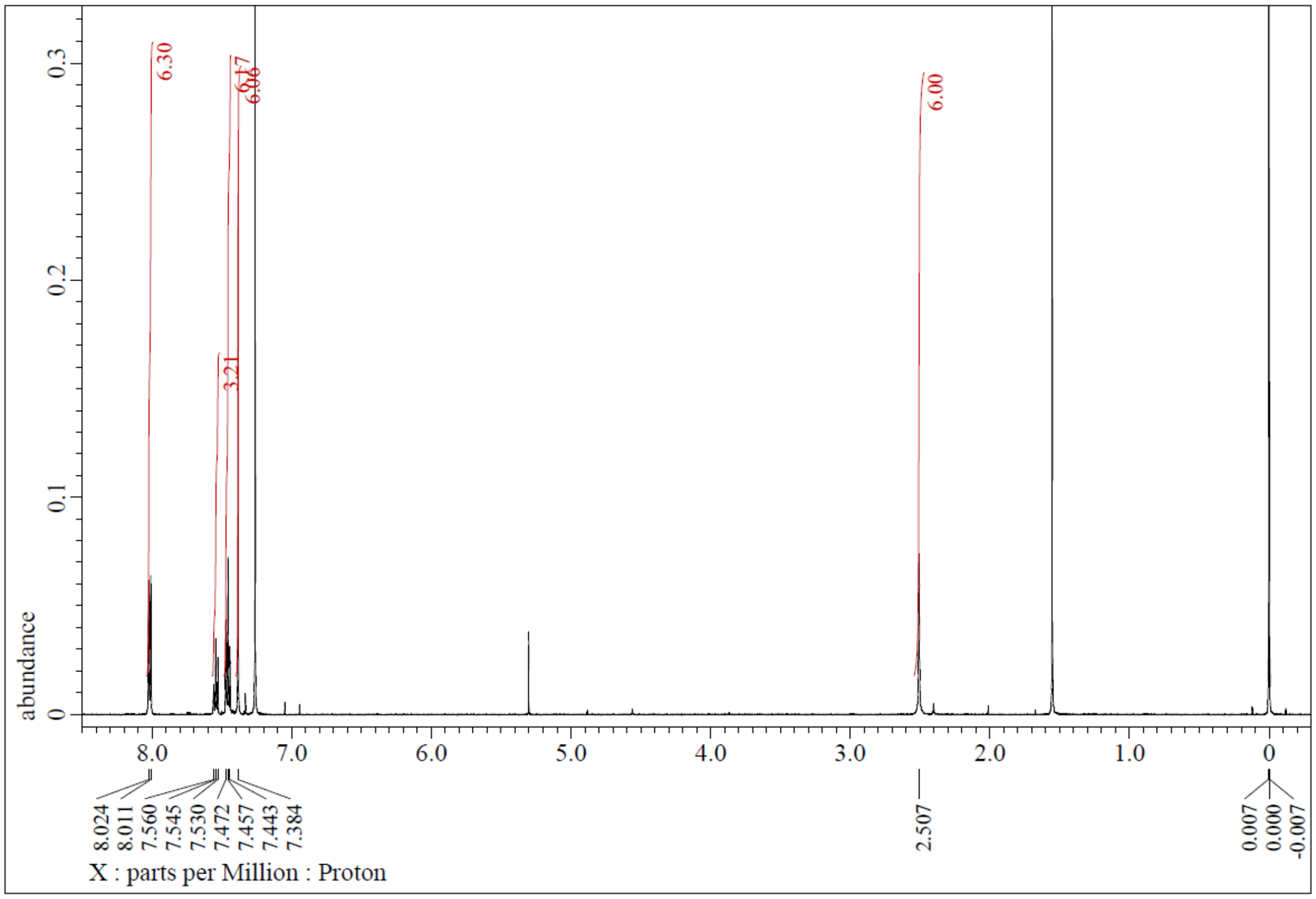

Figure S14. ^{1}H NMR (500 MHz, $CDCl_3$) spectrum of **TripPh-Me**.

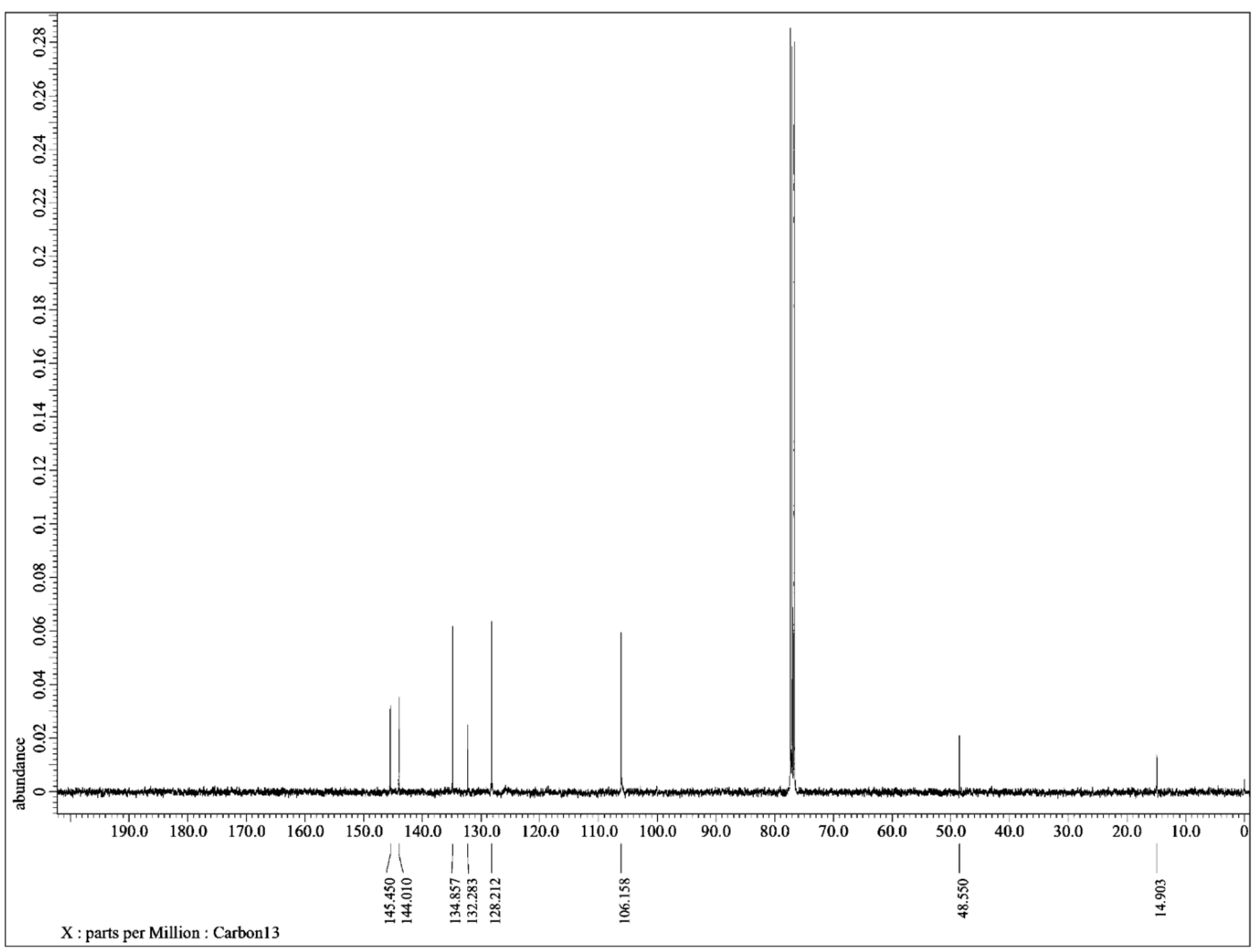

Figure S15. ^{13}C NMR (100 MHz, $CDCl_3$) spectrum of **TripPh-Me**.

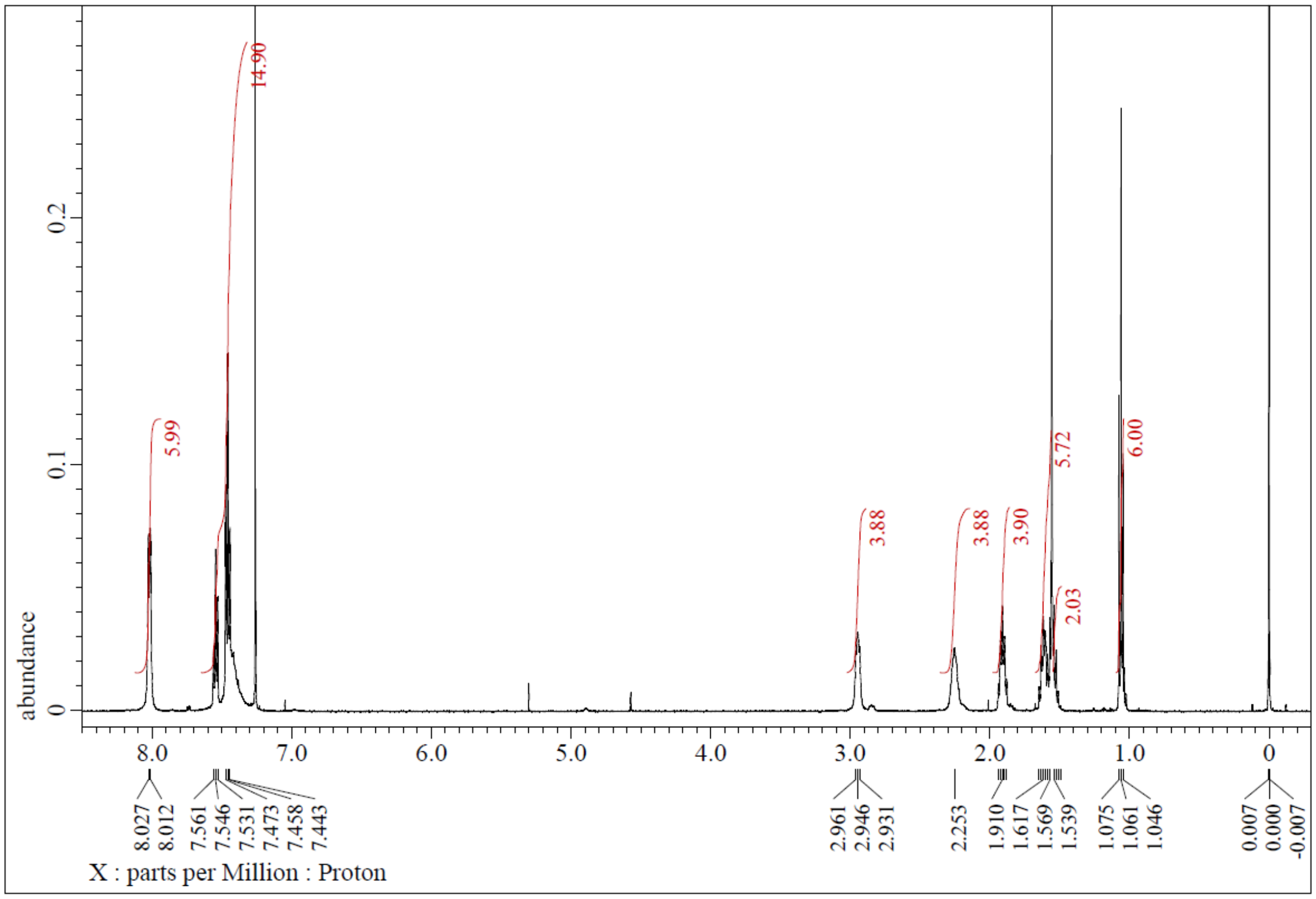

Figure S16. ^{1}H NMR (500 MHz, $CDCl_3$) spectrum of compound **TripPh-Hx**.

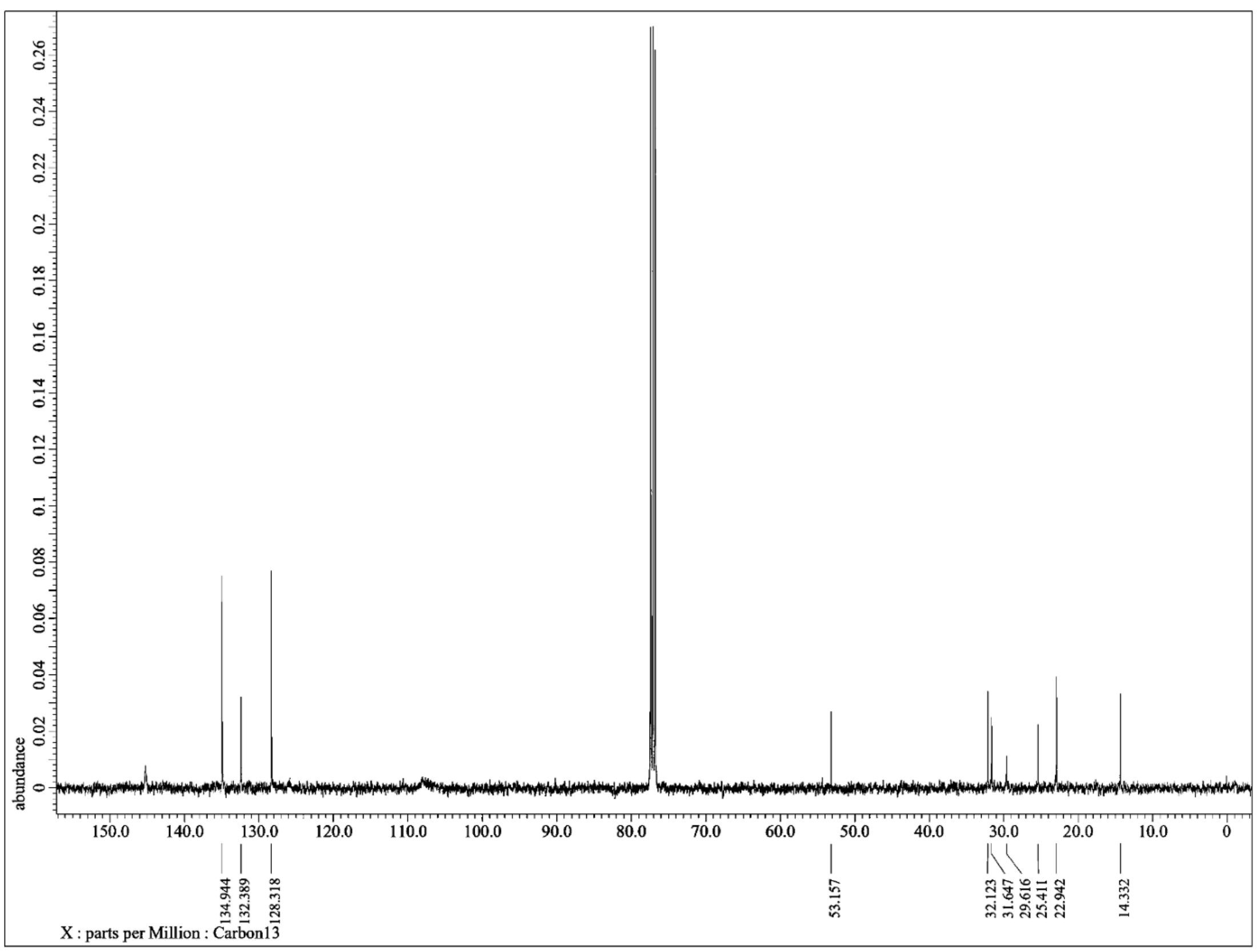

Figure S17. ^{13}C NMR (100 MHz, $CDCl_3$) spectrum of compound **TripPh-Hx**.

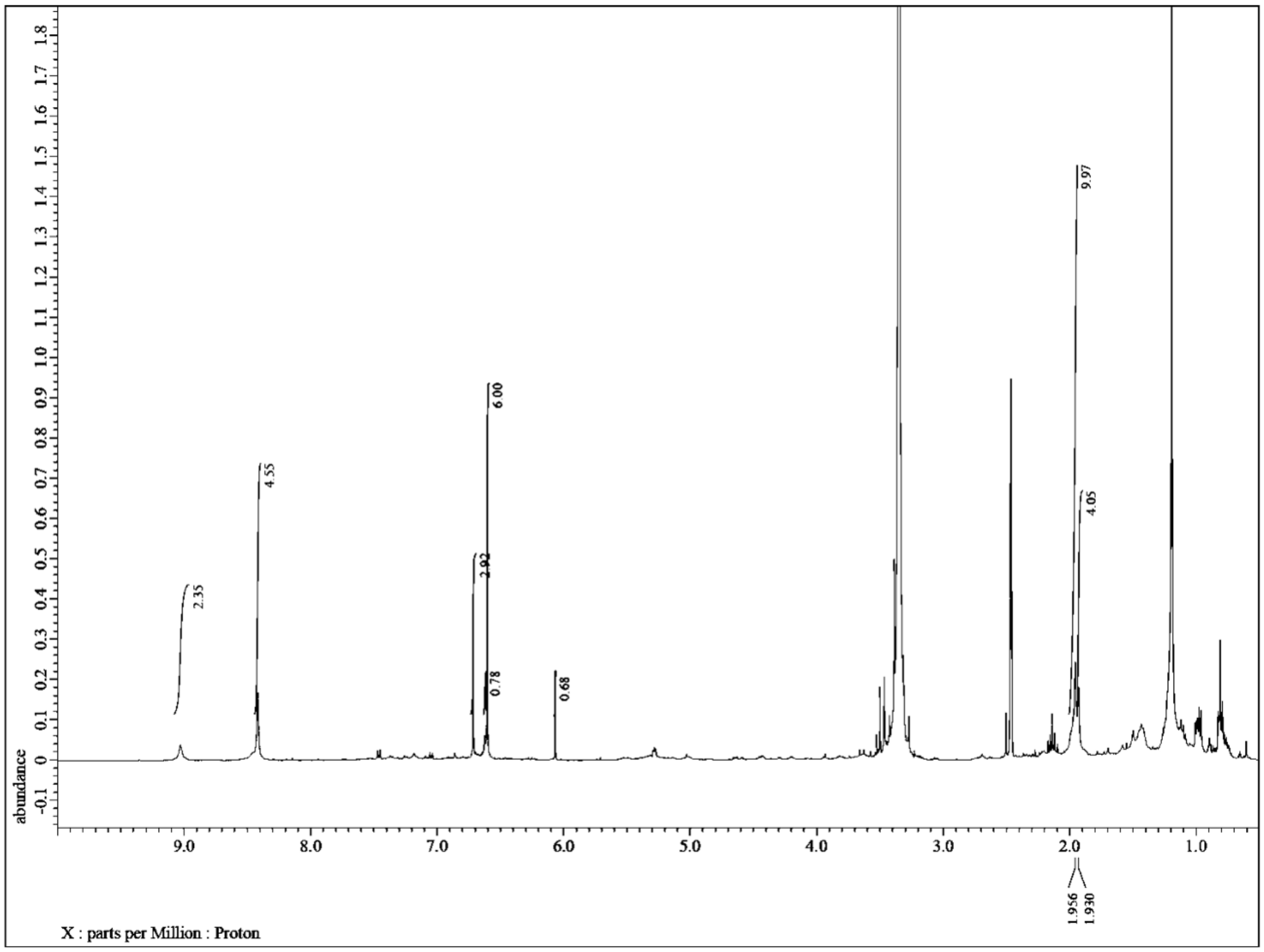

Figure S18. ^{1}H NMR (500 MHz, DMSO-d_6) spectrum of decomposed materials.

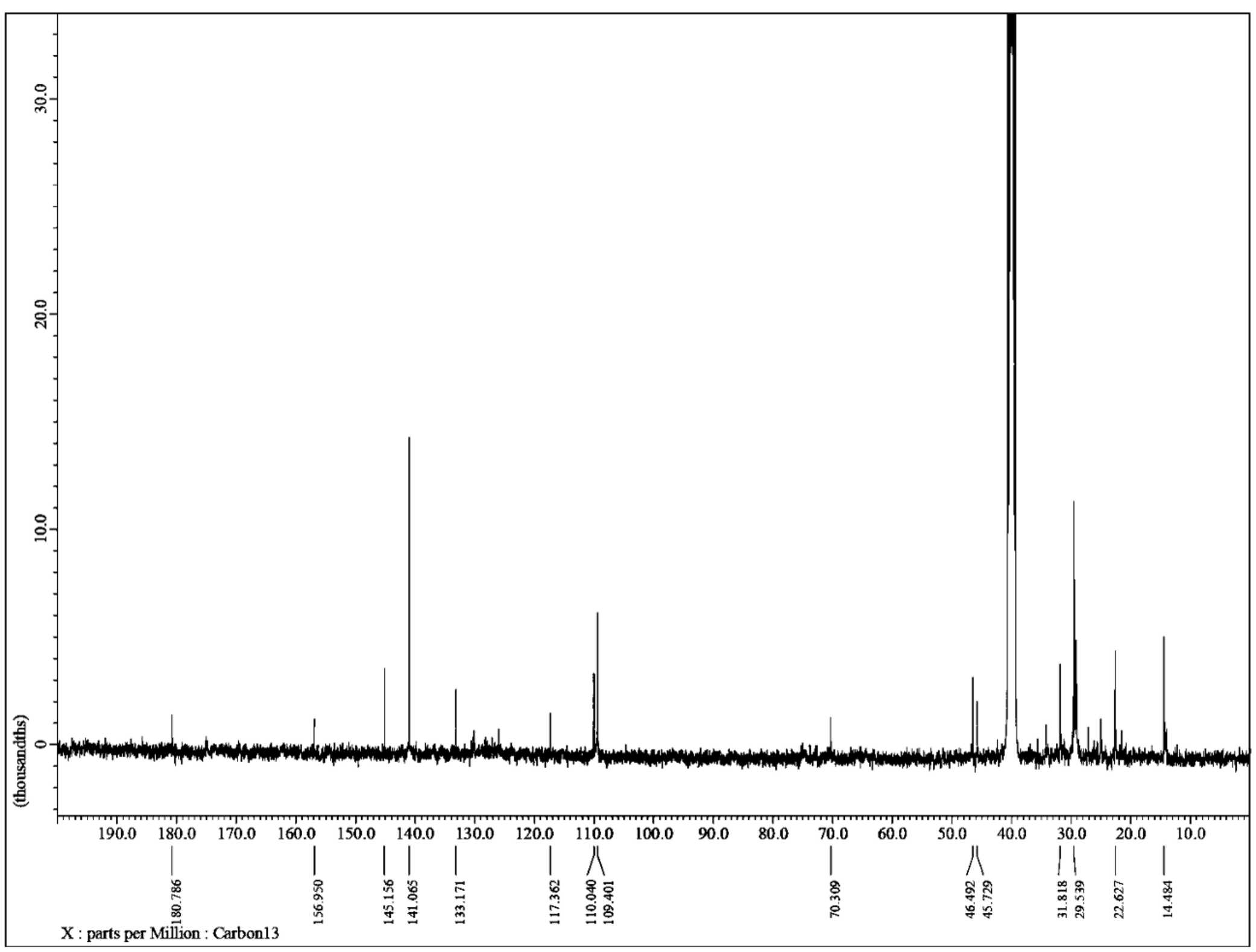

Figure S19. ^{13}C NMR (100 MHz, DMSO-d_6) spectrum of decomposed materials.

14. MS

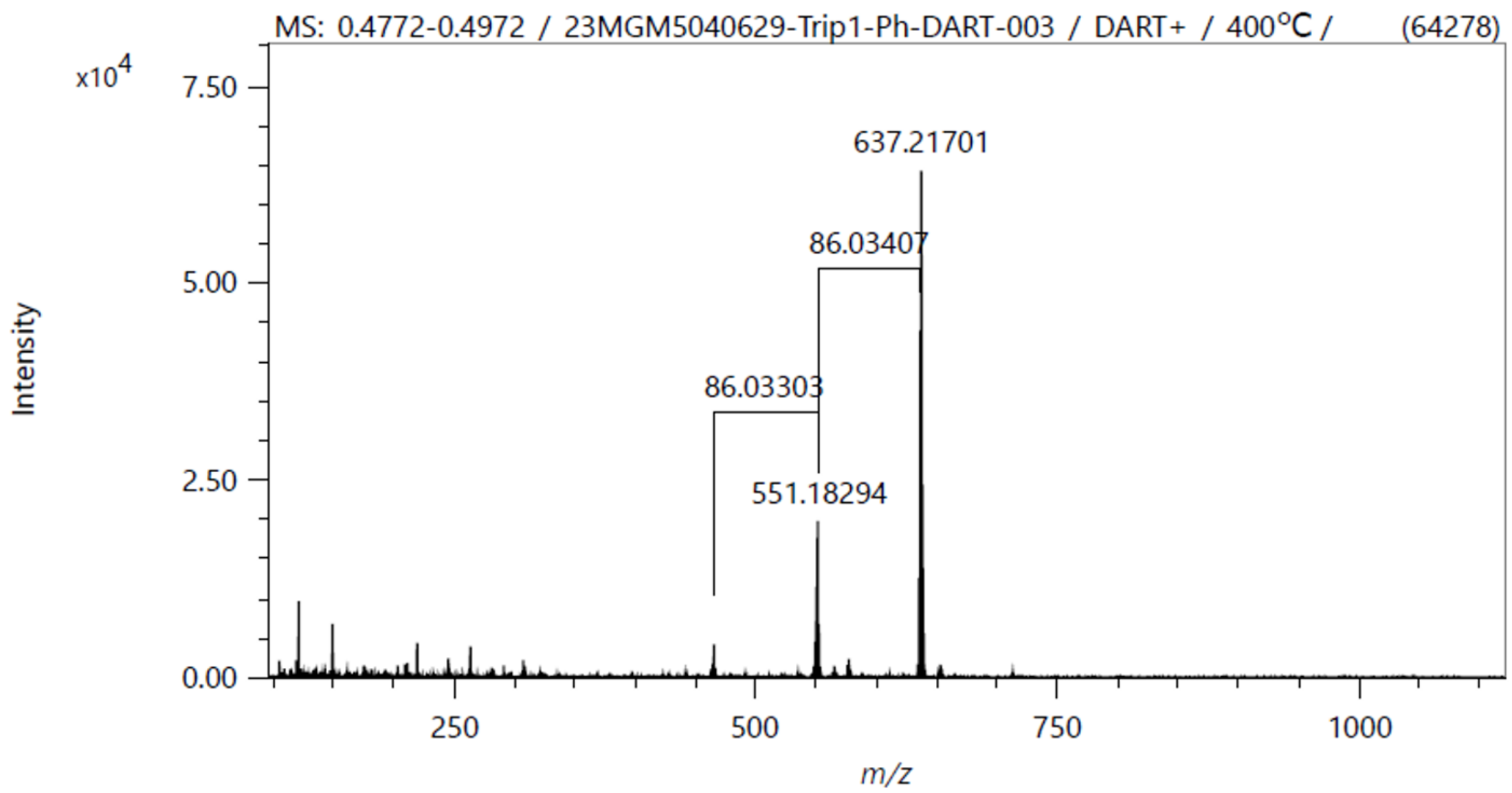

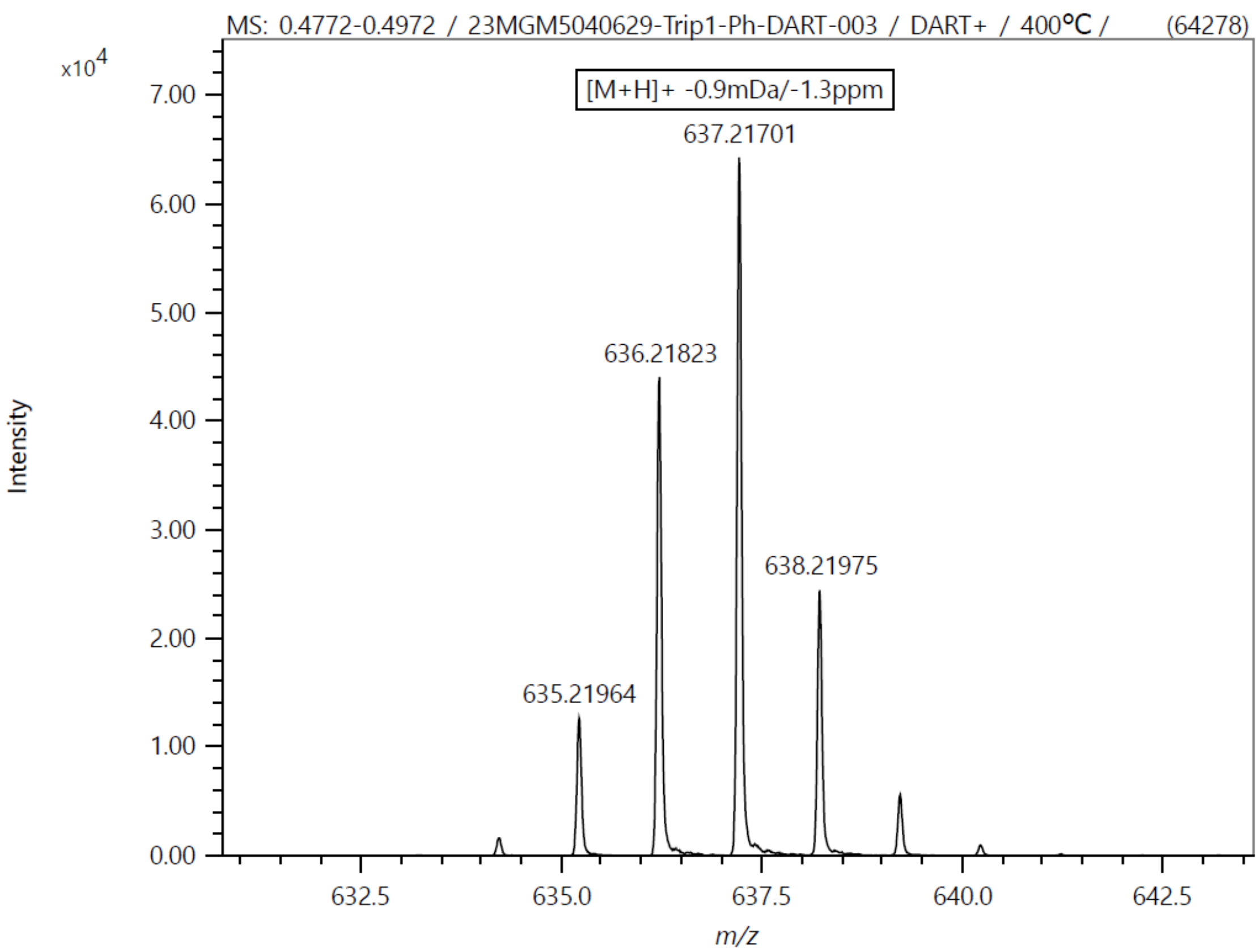

Figure S20. HR-DART mass spectrum of **TripPh-Me**.

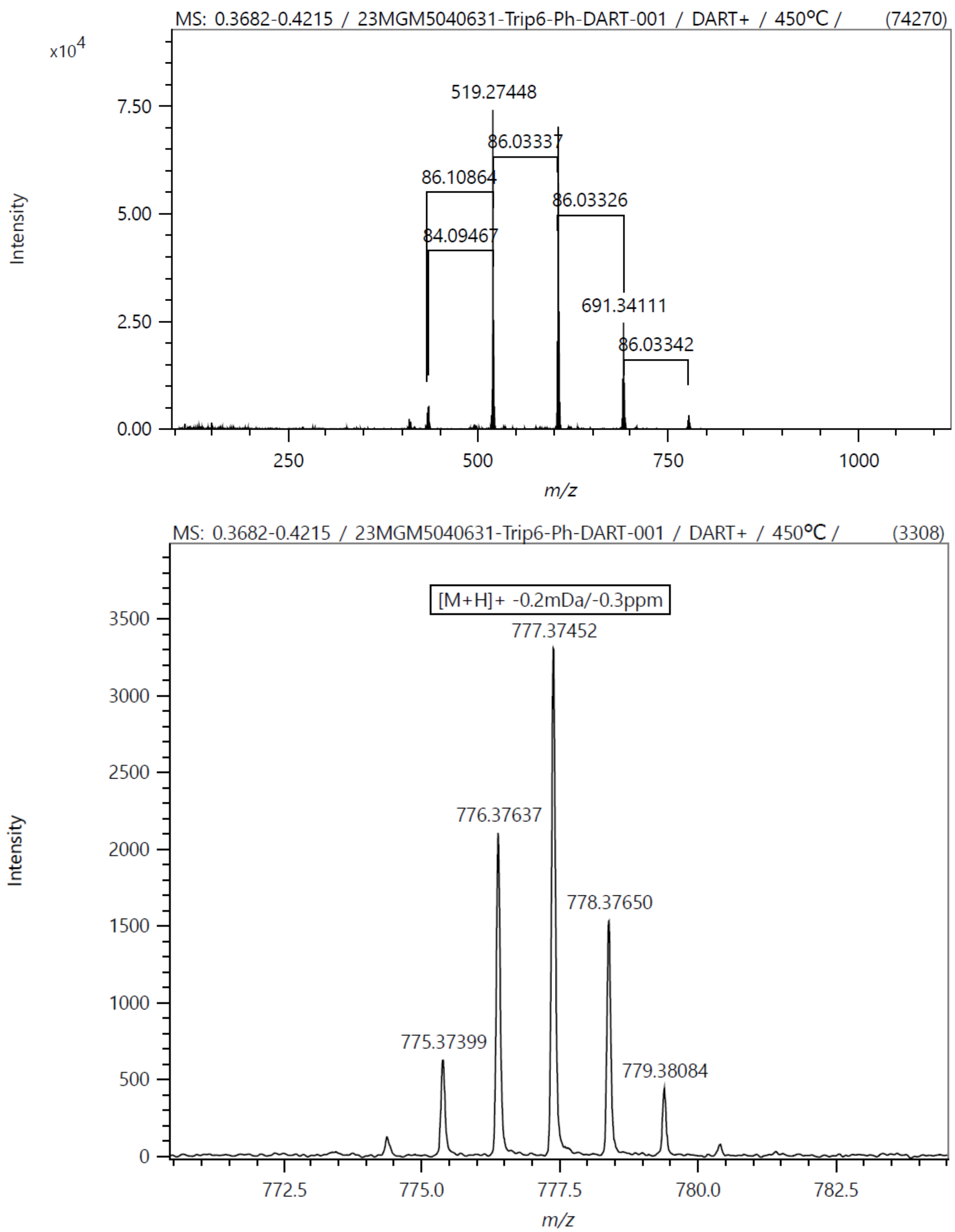

Figure S21. HR-DART mass spectrum of **TripPh-Hx.**
